# High Output Voltage Aqueous Supercapacitors by Water Deactivated Electrolyte over Wide Temperature Range

**DOI:** 10.1002/advs.202500385

**Published:** 2025-02-18

**Authors:** Hongji Wang, Wenpeng Liu, Jin Huang, Tianliang Xiao, Wenwei Lei, Faming Gao, Mingjie Liu

**Affiliations:** ^1^ School of Environmental and School of Environmental and Chemical Engineering Yanshan University Qinhuangdao Hebei 066004 China; ^2^ Key Laboratory of Bio‐inspired Smart Interfacial Science and Technology of Ministry of Education School of Chemistry Beihang University Beijing 100191 China

**Keywords:** high output voltage, inner Helmholtz plane, intermolecular interaction, supercapacitors, water deactivated organohydrogel electrolytes

## Abstract

Confined by factors such as low operating voltage, poor temperature resistance, and instability at high voltage, the energy density of conventional symmetric aqueous supercapacitors is undesirable over a wide temperature range. It is still challenging to develop aqueous flexible supercapacitors (AFSCs) that can provide stable and high voltage output (>2.0 V) at extreme ambient temperatures. Here, a strategy for constructing AFSC with ultrahigh output voltages over a wide temperature range is proposed through the development of organohydrogel electrolytes (OHEs) with excellent water deactivation, which achieve a notable output voltage of 3.0 V, and unprecedented energy densities of 23.16 µWh cm^−2^ at −40 °C (beyond 25 °C), surpassing the performance of all previously reported symmetric supercapacitors with aqueous electrolytes. Theoretical calculations and experimental analyses show that OHEs can deactivate water to increase the output voltage limit of AFSCs by enhancing intermolecular interactions and regulating inter Helmholtz plane. Meanwhile, it also shows excellent flexibility and cycling stability (80.5% after 20 000 cycles at 25 °C and 97.0% after 50 000 cycles at −40 °C). More importantly, OHEs enable AFSCs switchable output voltages (from 2.5 to 3.0 V), making it possible to operate supercapacitors with high energy density and stability at low temperatures.

## Graphical abstract

NMP‐regulated intermolecular interactions (formation of hydrogen bonds and solvation structures) and the inner Helmholtz plane deactivate water in the aqueous gel electrolyte and inhibit water decomposition over a wide temperature range, enabling aqueous flexible supercapacitors with output voltages as high as 3.0 V and energy densities exceeding room temperature.

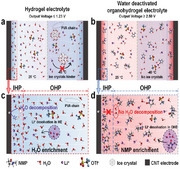

## 1 Introduction

The rapid advancement in flexible and wearable electronic devices is fueling the demand for rechargeable flexible energy storage that offers enhanced safety and stability, as well as high energy density.^[^ ^1^ ^]^ Aqueous supercapacitors featuring hydrogel electrolytes have emerged as prime candidates for powering flexible electronic devices owing to their numerous advantages, including lightweight design, high‐power density, excellent cycle reversibility, prolonged cycle life, and inherent safety.^[^ ^2^ ^]^ However, the energy density of AFSCs is constrained by the theoretical decomposition voltage of water, particularly evident at sub‐zero temperatures, where the energy density experiences significant attenuation, thereby limiting the applicability of flexible supercapacitors across a wide temperature range.^[^ ^3^ ^]^ Therefore, advanced electrolytes capable of increasing the output voltage of supercapacitors must be developed to address the challenge of low energy density.

The main challenge in extending the electrochemical stability window (ESW) of aqueous‐based electrolytes is to inhibit the water activity in the electrolyte and at the electrode interface to cross the water decomposition reaction.^[^ ^3a^ ^]^ This can be achieved by disrupting the hydrogen bonding network among the water within the electrolyte and reducing the water content in the solvation structure (hereafter collectively referred to as intermolecular interactions), thereby decreasing water activity and broadening the ESW,^[^ ^4^ ^]^ as well as impede the formation of ice crystals and increase the freezing resistance of the electrolyte.^[^ ^5^ ^]^ Current state‐of‐the‐art strategies involve using “water‐in‐salt” (WIS) electrolytes and organic cosolvents or additives.^[^ ^6^ ^]^ While ultrahigh concentrations of salt effectively disrupt the H‐bonding network and widen the ESW, the strong interactions between cations and anions result in high viscosity and low ionic conductivity of the electrolyte.^[^ ^7^ ^]^ Specifically, ionic conductivity experiences a sharp decline in sub‐zero environments, leading to poor low‐temperature performance. Conversely, organic cosolvents, particularly those with high dielectric constants and donor numbers (DNs), can selectively displace H_2_O molecules in the solvation sheaths, thereby reducing solvation water content and activity while maintaining high ionic conductivity.^[^ ^8^ ^]^ Recently, a series of high‐performance AFSCs have been developed, achieving an output voltage of 2.0 V by employing organic solvents such as ethylene glycol and dimethyl sulfoxide as cosolvents for aqueous electrolytes.^[^ ^9^ ^]^ However, reports of such high‐performance AFSCs are scarce, and to date, the output voltage remains below 2.5 V.

The electrode‐electrolyte interface serves as the primary site for water decomposition side reactions.^[^ ^10^ ^]^ Thus, facilitating the water deactivation at the electrode‐electrolyte interface is essential for further enhancing the output voltage and energy density of AFSCs. The inter Helmholtz plane (IHP) is nearest to the electrode within the electrolyte, where undergoes a desolvation process and subsequent specific adsorption that dictates the water decomposition reaction.^[^ ^11^ ^]^ Desolvated H_2_O molecules are readily adsorbed onto the electrode surface, initiating water splitting that hampers the expansion of the output voltage of supercapacitors.^[^ ^12^ ^]^ By restructuring the IHP through the addition of cosolvents, partially desolvated H_2_O molecules can be eliminated from the electrode surface while concurrently forming an H_2_O‐deficient solvated shell. This adjustment effectively reduces the proportion of H_2_O within the IHP, thus inhibiting the reactivity of the free H_2_O decomposition reaction. Consequently, regulating intermolecular interactions while modifying the IHP structure is crucial for increasing the high‐voltage limit of AFSCs by further inhibiting the water activity to suppress the decomposition reaction of H_2_O molecules.

**Scheme 1:**
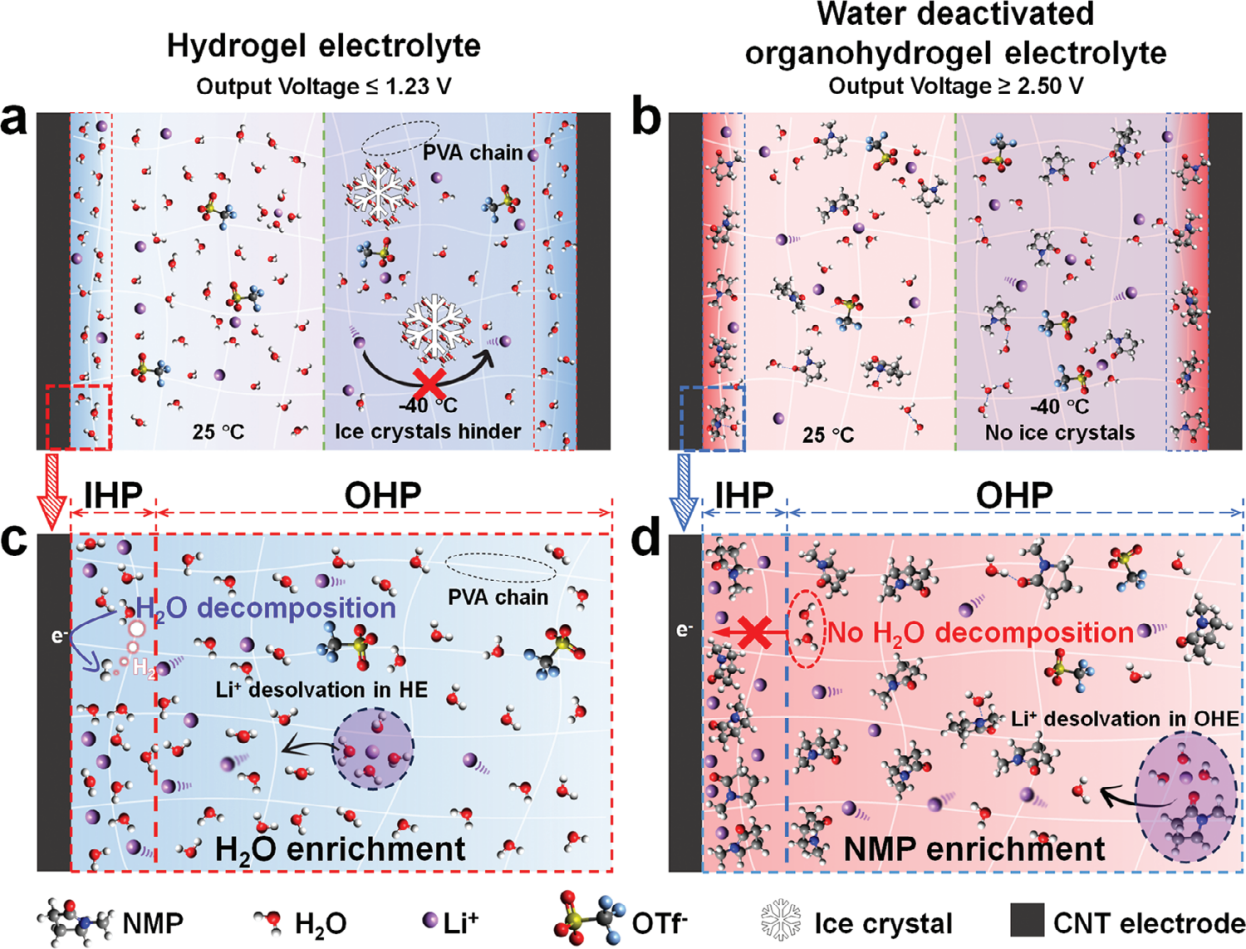
Design strategies for wide output voltage and low‐temperature‐resistant gel electrolytes. Illustration of internal molecule interaction in a) HE and b) Water deactivated OHE at temperatures of 25 °C and −40 °C, respectively. Illustration of desolvation at OHP (outer Helmholtz plane) and specific adsorption at the IHP of c) HE and d) OHE, respectively. Li, C, H, O, N, F, and S atoms are indicated by purple, gray, white, red, blue, indigo, and yellow, respectively. IHP and OHP represent the inner and outer Helmholtz planes, respectively. Compared with conventional HEs, OHEs enable high output voltage and low‐temperature resistance of supercapacitors by synergistically modulating intermolecular interactions (solvation structure and H‐bonding) and IHP.

In this study, water‐deactivated OHEs were prepared by immersing PVA organogel into a LiOTf water/N‐methylpyrrolidone (NMP) solution, aimed at constructing high‐voltage and low‐temperature‐resistant AFSCs. Compared with conventional hydrogel electrolytes (HEs), NMP is utilized as a cosolvent in OHEs to achieve excellent electrochemical performance (high output voltage and energy density) of supercapacitors over a wide temperature range (**Scheme** **1**). Water deactivation through synergistic regulation of intermolecular interactions and IHP structure by NMP, symmetrical AFSCs fabricated using OHE‐4.5 and carbon nanotube (CNT) electrodes exhibit a higher output voltage with 2.5 V at room temperature compared to all previously reported aqueous supercapacitors. Moreover, the synergistic effect is enhanced at low temperatures, further mitigating water decomposition. AFSC‐4.5 exhibits an impressive 3.0 V output voltage and outstanding stability, with capacity retention exceeding 95% after 50 000 charge/discharge cycles across the −25 to −40 °C temperature range. Owing to the elevated working voltage of AFSC‐4.5, its energy density increases by 110% at −25 °C and 105% at −40 °C compared to that at 25 °C. This ability to regulate the output voltage at varying temperatures enables high‐performance power, thereby enhancing the adaptability of supercapacitors and broadening their practical application.

## 2 Results and Discussion

### 2.1 Fabrication of Flexible Anti‐Freezing OHEs with Wide ESWS

**Figure 1:**
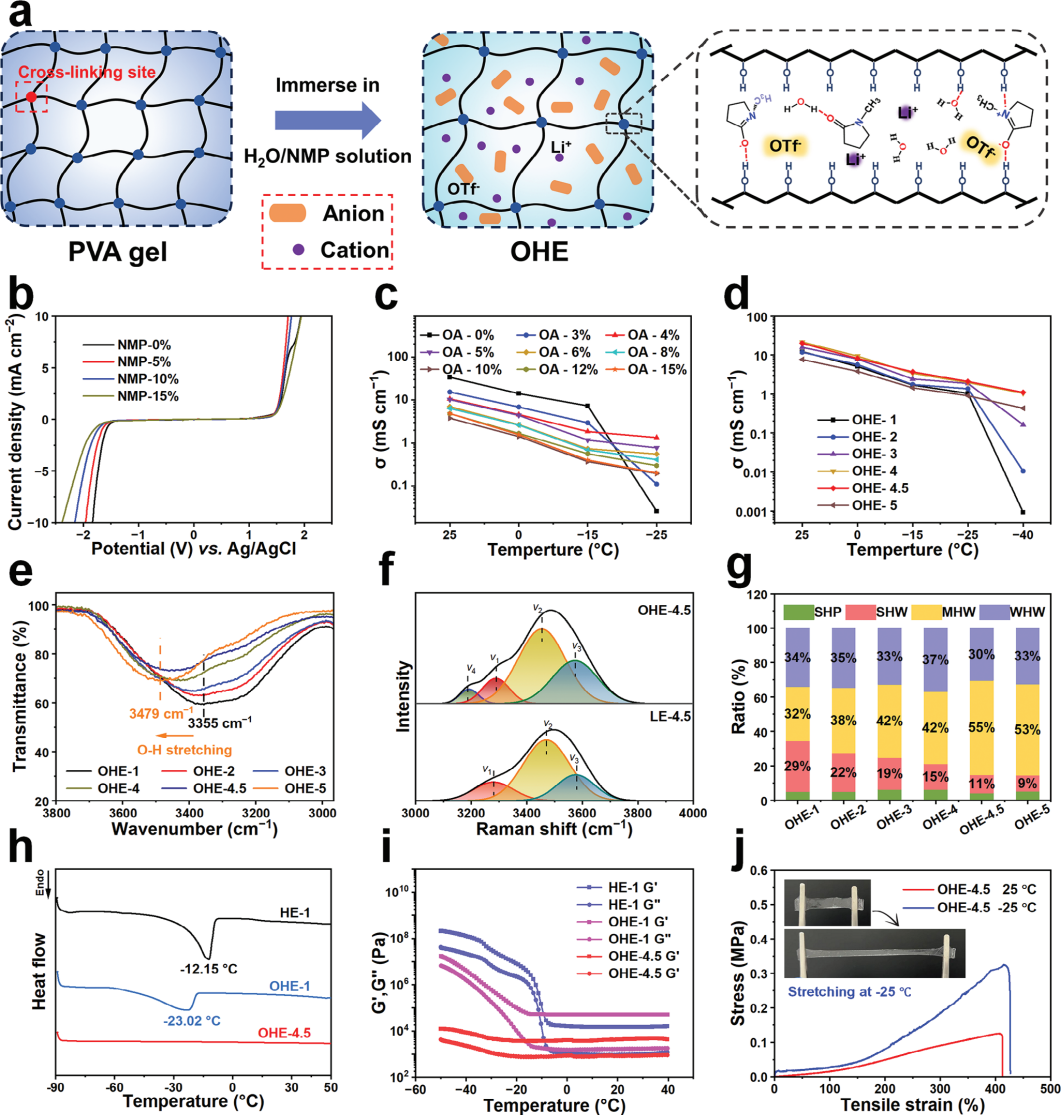
Preparation process and physical properties of OHEs. a) Preparation of gel electrolyte. b) ESW for various LEs with different contents of NMP determined by LSV tests on three electrodes system at a scan rate of 5 mV s^−1^ from −3.0 to 3.0 V versus Ag/AgCl electrode. c) Ionic conductivity (σ) of gel electrolytes under the temperature range from 25 to −25 °C with different contents of NMP. d) Ionic conductivity of OHEs under the temperature ranges from 25 to −40 °C with different concentrations of LiOTf. e) FTIR spectra of OHEs in 3800–2965 cm^−1^ region. f) Raman spectra peak fitting of LE‐4.5 and OHE‐4.5 in 3000–4000 cm^−1^ region (v_1_: SHW, v_2_: MHW, v_3_: WHW, v_4_: PHW). g) Proportion of H‐bonding interactions with water molecules in OHEs. h) DSC curves of HE‐1, OHE‐1 and OHE‐4.5 with temperature from −90 to 50 °C. i) Storage moduli (G′) and loss moduli (G″) of gel electrolytes from 40 to −50 °C. j) Tensile stress–strain curves at 25 and −25 °C of OHE‐4.5.

**Figure** **1**a depicts a flexible polyvinyl alcohol (PVA) organogel precursor that was prepared through the methodology outlined in our previous work,^[^ ^13^ ^]^ as illustrated in the Experimental Section of the Supporting Information. Subsequently, the OHE was synthesized by immersing the PVA organogel in a solution comprising water/NMP and LiOTf. NMP has desirable properties such as low melting point, high boiling point, strong polarity, high dielectric constant, and DN. First, linear sweep voltammetry (LSV) tests were conducted on liquid electrolytes (LEs) containing 1 M LiOTf and varying NMP concentrations to examine the impact of NMP as a cosolvent on the ESW (Figure 1b). An increase in the molar fraction of NMP significantly broadened the ESW of the electrolyte, indicating that the cosolvent NMP can reduce water activity in the electrolyte and thus inhibit water decomposition. However, similar to other organic cosolvents, NMP adversely affects the ionic conductivity of the electrolyte.^[^ ^14^ ^]^ Therefore, the addition ratio must be carefully regulated to ensure optimal electrolyte conductivity across different temperatures. The organogels were immersed in different mixtures of 1 M LiOTf aqueous electrolyte and NMP to fabricate OHEs (denoted as OA‐x%, where x represents the molar fraction of NMP in the mixture solution). As illustrated in Figure 1c, at temperatures of 25 °C to −15 °C, OA‐0% exhibited higher ionic conductivity compared to OHEs with NMP additives. However, at −25 °C, the ion conductivity of OA‐0% experienced a steep decline, while OA‐4% demonstrated the highest ionic conductivity, reaching 1.34 mS cm^−1^ at −25 °C and remaining at 10.65 mS cm^−1^ at 25 °C. To further extend the operating temperature range of OHEs, the salt concentration was adjusted based on OA‐4%. The increase in ionic conductivity with salt concentration from 1 to 4.5 m, is due to the fact that more ions in the electrolyte are well dispersed in the solvent, and more ions are able to be transported rapidly through the electrolyte, thus increasing the ionic conductivity of the electrolyte. However, with further increase up to 5 m, the interaction between the anions and cations, which slows down the transport of ions, leads to a decrease in the ionic conductivity. (Figure 1d; FigureS1, Supporting Information).^[^ ^7^ ^]^

Fourier transform infrared (FTIR) and Raman spectroscopy were employed to elucidate the molecular interactions within OHEs. It was observed that with increasing NMP content, the O−H peaks in water molecules shifted to higher wavenumbers, while the stretching vibrations of the C─N and C═O peaks in the NMP molecule shifted to lower and higher wavenumbers, respectively (Figure S2a,b, Supporting Information). This phenomenon suggests that new hydrogen bonds were formed between NMP and water molecules, thus disrupting the original H‐bonding network among highly activated water molecules.^[^ ^4^, ^12^ ^]^ As illustrated in Figure S2c,d (Supporting Information) similar peak position shifts of NMP and water molecules were consistent between the LEs and OHEs. FTIR analysis of OHEs revealed gradual shifts of characteristic O─H peaks to higher wavenumbers accompanied by weakening with increasing salt concentration (Figure 1e). Similarly, the O─H stretching vibration of LE and OHE shifted to higher wavenumbers in the 3000–3800 cm^−1^ (Figure S3, Supporting Information). Raman spectra with increasing salt concentration indicate greater involvement of water molecules in dissolution. To investigate the effect of salt on water molecules in electrolytes, the O─H stretching vibration of water molecules in LEs typically deconvolved into three components: strong hydrogen‐bonded water (SHW), medium hydrogen‐bonded water (MHW), and weak hydrogen‐bonded water (WHW).^[^ ^15^ ^]^ As depicted in Figure 1f, unique hydrogen bonds formed by the presence of PVA chains in OHEs (PHW) were also revealed. The O−H peaks of OHEs at different concentrations were deconvolved to determine the proportions of different types of hydrogen bonds (Figure S4, Supporting Information). It was observed that SHW content gradually decreased, indicating increased formation of hydrogen bonds or solvation structures with NMP molecules or salts (Figure 1g), thereby reducing water molecule activity and enhancing the low‐temperature resistance of OHEs. These findings collectively demonstrate that modulation of NMP and salt content can disrupt the intramolecular H‐bonding network and inhibit water reactivity, thus significantly contributing to broadening the ESW and enhancing the low‐temperature ionic conductivity of the electrolyte.^[^ ^16^ ^]^

Optical photographs in Figure S5 (Supporting Information) depict the varying states of OHEs at different concentrations. At a concentration of 4.5 m, the gel retains its initial organogel size and exhibits non‐flammability. However, excess ions within OHE‐5 disrupt cross‐linking sites, resulting in rupture and poor mechanical properties. Subsequently, the LSV curves of different OHEs were tested with the Swagelok system and the ESW of the OHEs increased from 1.14 to 3.17 V, which is expected to broaden the output voltage of flexible supercapacitors (Figure S6, Supporting Information). Additionally, differential scanning calorimetry (DSC) measurements show a distinct peak corresponding to the melting of ice at −12.15 and −23.02 °C for HE‐1 and OHE‐1, respectively, while no peak was detected for OHE‐4.5, even at −90 °C, which revealed that OHE‐4.5 has better low‐temperature resistance (Figure 1h; Figure S7, Supporting Information). Rheological and stress‐strain tests further confirmed the low‐temperature resistance and excellent flexibility of OHE‐4.5 over a wide temperature range (Figure 1i,j). Consequently, considering the mechanical properties, safety, low‐temperature resistance, and ESW of OHEs, OHE‐4.5 was selected for supercapacitor assembly and subsequent investigation of its electrochemical properties.

### 2.2 Electrochemical Performance of the AFSC over Wide Temperature Range

**Figure 2:**
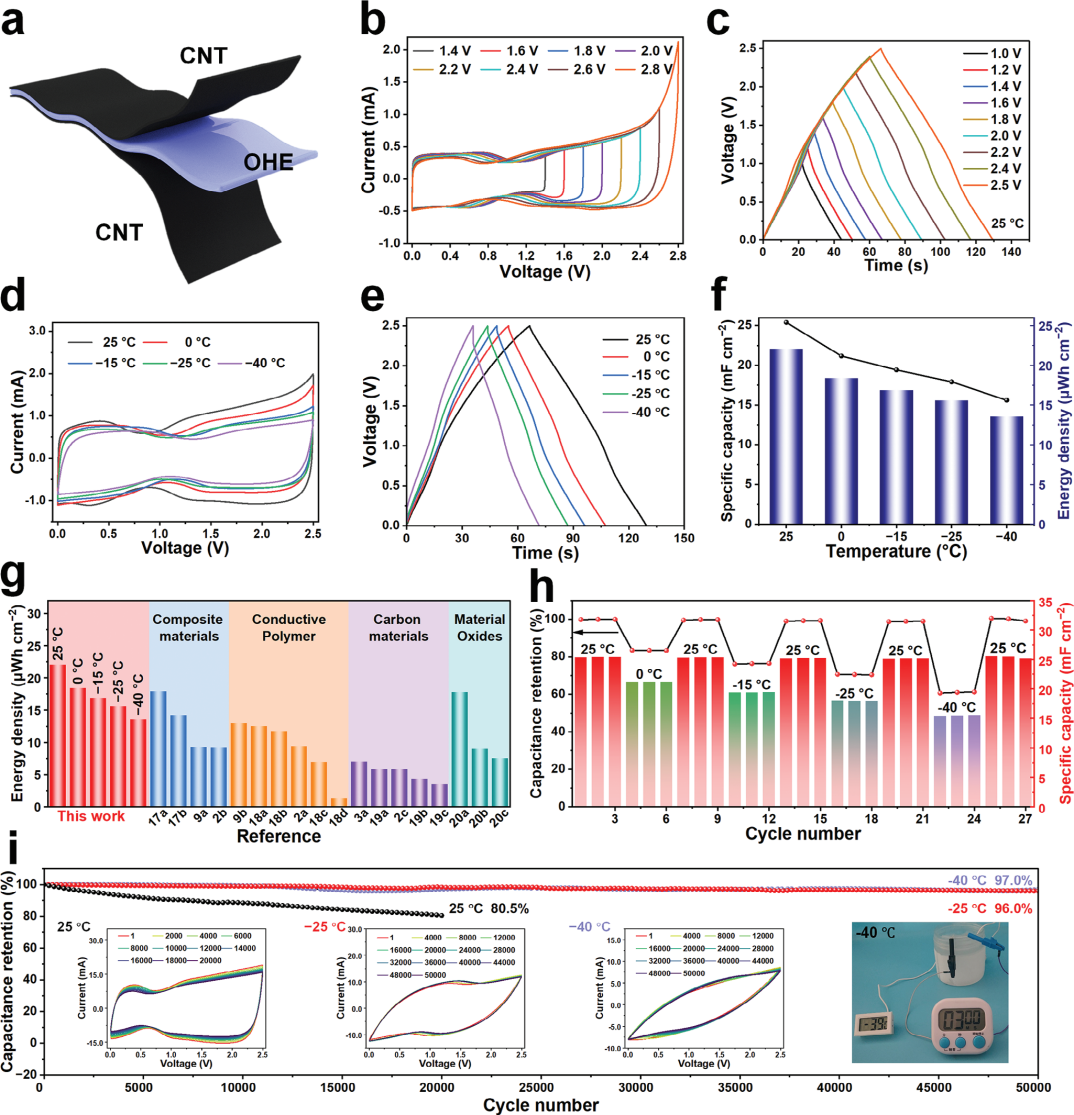
Electrochemical performance of AFSC‐4.5 over a wide temperature range. a) Structure of flexible supercapacitor. b) CV curves of AFSC‐4.5 in voltage ranges of 1.0 to 2.8 V at a scan rate of 10 mV s^−1^. c) GCD curves of AFSC‐4.5 in voltage ranges of 1.0 to 2.5 V at 1.0 mA cm^−2^. d,e) CV and GCD curves of AFSC‐4.5 at different temperatures. f) Specific capacitance and energy density of AFSC‐4.5 at different temperatures. g) Comparison of the energy density of AFSC‐4.5 with other high‐performance supercapacitors reported previously. h) Continuous reversible changes of capacitance retention of AFSC‐4.5 at different temperatures. i) Cycling stability of AFSC‐4.5 measured at 500 mV s^−1^ within the voltage window of 0–2.5 V at different temperatures (The insets show the CV curves at different temperatures for different number of cycles at 2.5 V and AFSC‐4.5 powering a timer at −40 °C).

As illustrated in **Figure** **2**a, OHE‐4.5 serves as the electrolyte and is attached to both sides with CNT to form a flexible “sandwich” supercapacitor (AFSC‐4.5). Cyclic voltammetry (CV) testing was employed to determine the output voltage of AFSC‐4.5 at 25 °C (Figure 2b). Notably, a significant polarization of the CV curve occurred at voltages above 2.6 V, indicating the possible presence of a water decomposition reaction. In addition, the effect of the optimized electrolyte process on deactivated water was studied by comparing the CV curves of different gel electrolytes in the range of 0–2.5 V. OHE‐4.5 has almost no polarization phenomenon, and has higher electrochemical stability than HE‐4.5 and other OHEs (Figure S8, Supporting Information). Increased water deactivation can effectively broaden the output voltage of the AFSCs. Therefore, to avoid the decomposition reaction affecting the stability of the supercapacitor under high voltage, choose a voltage range of 0–2.5 V. Galvanostatic charge‐discharge (GCD) was used to test the properties of AFSC‐4.5, the GCD curves of AFSC‐4.5 exhibit symmetrical triangles under different voltages, indicating good capacitive performance (Figure 2c). Compared to 1.0 V, the marked difference in energy density of AFSC‐4.5 at 2.5 V (increased by ≈7 times) underscores the advantage of an extended voltage range (Figure S9, Supporting Information). Additionally, in Figure S10a (Supporting Information), CV tests at different scan rates within the voltage range of 0–2.5 V display an approximately rectangular shape, indicating the capability of AFSC‐4.5 for rapid charge/discharge. The specific capacity of AFSC‐4.5, calculated from GCD curves, was 25.40 mF cm^−2^ at a current density of 1.0 mA cm^−2^ (Figure S10b, Supporting Information). Even as the current density increased to 10.0 mA cm^−2^, its specific capacitance remained high at 22.14 mF cm^−2^, corresponding to 78.79% retention of capacitance at 0.2 mA cm^−2^ (Figure S10c, Supporting Information), indicative of excellent fast charge/discharge performance. Owing to the outstanding flexibility of OHEs and CNT electrodes, AFSC‐4.5 maintains stable specific capacity at various bending angles (Figure S11a, Supporting Information). After 1200 bending cycles from 0 to 180°, AFSC‐4.5 retains a capacity retention rate of 98% (Figure S11b, Supporting Information). Subsequently, CV and GCD curves of multiple devices connected in series/parallel were tested. The ultrahigh output voltage enables the device to fulfill power requirements for numerous electronic devices with minimal integration. As depicted in Figure S11c,d (Supporting Information), when three devices are connected in series, the voltage can reach 7.5 V without any loss of specific capacity, and the capacity of three devices connected in parallel can be tripled.

The electrochemical performance of AFSC‐4.5 was evaluated under various temperatures (Figure 2d–f; Figure S12, Supporting Information). As depicted in Figure 2d,e AFSC‐4.5 maintains an ultrahigh output voltage of 0–2.5 V, exhibiting nearly rectangular CV curves and symmetrical triangular GCD curves within the temperature range of −40 to 0 °C. Comparative to 25 °C, AFSC‐4.5 demonstrated exceptional capacitance retention of 83.39% (21.18 mF cm^−2^), 76.50% (19.43 mF cm^−2^), 70.59% (17.93 mF cm^−2^), and 61.46% (15.61 mF cm^−2^) at 0, −15, −25, and −40 °C, respectively (Figure 2f). AFSC‐4.5, with its ultrahigh output voltage, yields an energy density exceeding 13.55 µWh cm^−2^ across the temperature range of −40 to 25 °C. Compared to flexible supercapacitors utilizing other electrode materials such as composite materials,^[^ ^2^, ^9^, ^17^ ^]^ conductive polymers,^[^ ^2^, ^9^, ^18^ ^]^ carbon materials,^[^ ^2^, ^3^, ^19^ ^]^ and metal oxides^[^ ^20^ ^]^ reported in the literature (Figure 2g; Table S1, Supporting Information), AFSC‐4.5 exhibits outstanding energy density over a wide temperature range. Figure S12 (Supporting Information) provides detailed CV (different scan rates) and GCD (different current densities) curves of AFSC‐4.5 at 0, −15, −25, and −40 °C, along with its high‐capacity retention at various current densities, indicating favorable reliability of our devices at low temperatures. Subsequently, the AFSC‐4.5 alternated between sub‐zero and 25 °C, resembling operational conditions during temperature fluctuations (Figure 2h). Its excellent low‐temperature performance mitigates the impact of temperature changes, endowing AFSC with exceptional environmental adaptability. Furthermore, it exhibited a high capacitance retention of 80.5%, 96.0%, and 97.0% after 20 000, 50 000, and 50 000 cycles at 25, −25, and −40 °C, respectively (Figure 2i). Similar stability was also demonstrated in the GCD test with 88.17% and 96.72% at 25 and −25 °C, respectively, proving that the AFSC‐4.5 has excellent cycling stability over a wide voltage range of 0−2.5 V (Figure S13, Supporting Information). Additionally, leveraging its high output voltage and energy density, AFSC‐4.5 successfully illuminated the LED lights with the pattern “YSU” at 25 °C and powered the electronic clock at different temperatures (the inset of Figure 2i; Figure S14, Supporting Information).

### 2.3 Mechanism of Water Deactivation for High Output Voltage

**Figure 3:**
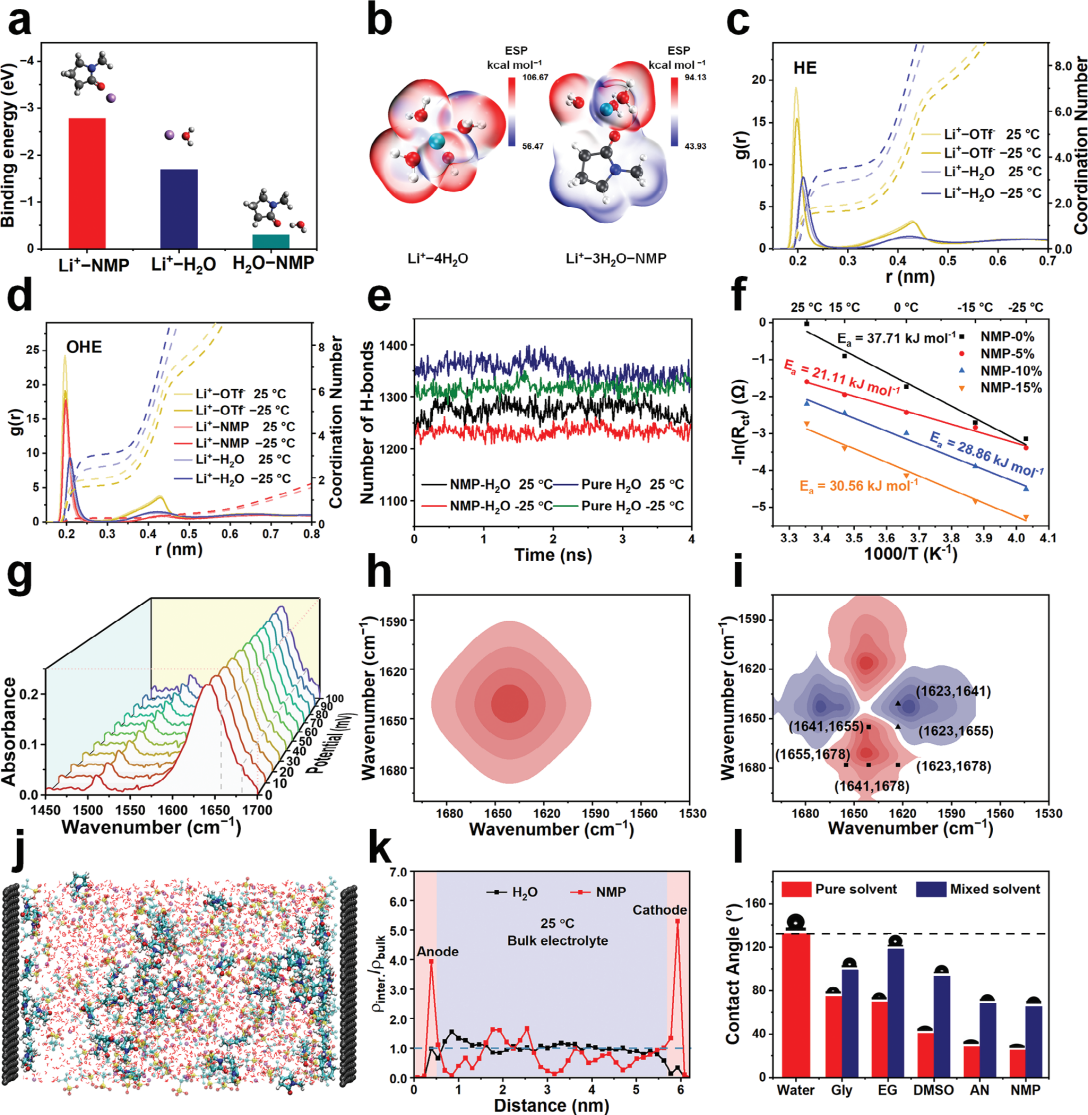
Mechanism in OHEs with high‐voltage and low‐temperature resistance. a) Binding energies of Li^+^─NMP, Li^+^─H_2_O, and H_2_O─NMP and corresponding structures. b) Electrostatic potential of a simple solvation model. The RDFs and CN of Li^+^─O were collected from the c) HE and d) OHE models. e) Number of hydrogen bonds between water molecules in the two systems at different temperatures. f) Desolvation‐free energy of electrolytes with different NMP contents. g) In situ FTIR spectra in regions for the NMP with increasing potentials on the working electrode as the perturbation. h) Synchronize and i) asynchronous 2D correlation spectra for the NMP in 1600–1680 cm^−1^ region. j) Snapshots of the simulated structures, including CNT under analog voltage. k) Density distributions of NMP and H_2_O in OHE at 25 °C. l) Contact angles of different solvents on the CNT surface before and after mixing with water (the proportion of co‐solvent in each of the solvent mixtures was 4 mol%).

The intrinsic mechanism of water deactivation for high output voltage and excellent low‐temperature performance of AFSC is elucidated by theoretical calculations. Density functional theory (DFT) was utilized to compute the binding energy between Li^+^ and solvent molecules. As depicted in **Figure** **3**a, the minimum binding energy value of Li^+^─NMP is −2.78 eV, indicating a preference for Li^+^ to bind with NMP and participate in the solvation structure. Therefore, two simplified solvation structural models, Li^+^─4H_2_O and Li^+^─3H_2_O─NMP, were constructed to calculate the electrostatic potential (ESP) distribution of the two solvation models. The Li^+^─3H_2_O─NMP model exhibited a smaller overall electrostatic potential, suggesting that the addition of NMP stabilizes the reconstituted solvation sheath while reducing the water molecules in the solvation structure and inhibiting water reactivity (Figure 3b).^[^ ^12^ ^]^ Furthermore, the radial distribution functions (RDFs) and coordination numbers (CNs) of HE and OHE simulation models (Figure S15, Supporting Information) were investigated by molecular dynamics (MD) simulation, and the solvated structures were analyzed at −25 and 25 °C (Figures 3c,d), respectively. Comparing the coordination radius of NMP with H_2_O, the smaller Li^+^─NMP radius in the Li^+^ solvation sheath indicates a stronger affinity between NMP and Li^+^, making NMP more likely to participate in solvation than H_2_O molecules.^[^ ^21^ ^]^ The CN of water molecules without/with NMP was reduced from 2.91 to 2.51, indicating that a small amount of NMP molecules effectively reduces the solvation sheath H_2_O content and achieves the purpose of reducing water activity.^[^ ^16^ ^]^ Additionally, with the decrease in the simulation temperature from 25 to −25 °C, both the CN of Li^+^─OTf^−^ in HE (from 1.93 to 1.66) and OHE (from 1.97 to 1.64) systems decrease due to the enhanced interaction of Li^+^─NMP and Li^+^─H_2_O (Figure S16, Supporting Information). This alteration in intermolecular interaction effectively impedes the agglomeration of anions and cations at low temperatures, enhancing ion transport performance. Simultaneously, the CN of H_2_O molecules also undergo significant changes (from 2.91 to 3.55 in HE and from 2.51 to 3.08 in OHE), indicating increased participation of H_2_O molecules in solvation, which further disrupts the H‐bonding network. This outcome is corroborated by the number of hydrogen bonds between water molecules. With the addition of NMP and lowering of temperature, the number of hydrogen bonds between water molecules within the electrolyte decreases (Figure 3e). Overall, these theoretical calculations demonstrate that the introduction of NMP effectively alters the solvation structure, reduces solvated water, and disrupts the H‐bonding network, which leads to the water deactivation in the decomposition reaction to afford high‐voltage antifreeze OHEs at wide temperature range. Furthermore, NMP inevitably affects the desolvation process and specific adsorption at the IHP. Initially, the desolvation‐free energy was estimated by fitting electrochemical impedance spectroscopy of different electrolytes at various temperatures (Figure 3f; Figure S17, Supporting Information). Among these, NMP‐5%, with a smaller amount of NMP, exhibits a minimum desolvation energy barrier of 21.11 kJ mol^−1^, indicating that Li^+^ can more easily be removed from the solvation structure, leading to better low‐temperature performance.

The desolvation process of Li^+^ at the IHP was further analyzed by applying a voltage to the OHEs to obtain in situ FTIR and 2D infrared correlation spectroscopy (2D IR COS), which is an analytical technique for various spectroscopic studies of complex systems. This method simplifies complex spectra with many overlapping peaks, enhances spectral resolution in 2D maps, and determines the sequence of spectral changes.^[^ ^22^ ^]^ Comparing the FTIR during voltage application, the C═O stretching vibration peak of NMP appears with several distinct peaks (1655 and 1678 cm^−1^), indicating interaction changes of NMP under voltage perturbation. As the voltage increases, the overall intensity of the peak increases, suggesting an increase in the NMP content near the electrode under the action of the electric field (Figure 3g; Figure S18, Supporting Information). Analysis of the synchronous and asynchronous 2D IR COS in 1750–1550 cm^−1^ region reveals the sequence of energy band intensity changes during the desolvation process (Figure 3h,i; Figure S19, Supporting Information): 1641 cm^−1^ (solvated NMP) →1655 cm^−1^ (H‐bond NMP) →1623 cm^−1^ (solvated water) →1678 cm^−1^ (free NMP), indicated as 1) NMP is perturbed by voltage under actual conditions, preferentially desolvated, and enriched in the IHP, preventing water from contacting the electrode; 2) NMP forms hydrogen bonds with free water molecules and desolvated water molecules, reducing the activity of water molecules in the IHP.

After desolvation of the electrolyte at the IHP, solvent molecules undergo specific adsorption on the electrode surface. The impact of charged electrodes in OHEs on the IHP within the electrode surface was assessed through MD simulations using a graphene electrode as a model system. As illustrated in Figure 3j, under slight potential perturbation, during the NMP‐dominated desolvation process, NMP molecules are adsorbed and enriched on the electrode surface. By contrast, active H_2_O molecules are expelled from the IHP, thus inhibiting contact between the water molecules and the electrode. The normal distribution of NMP and H_2_O along the electrode surface in the simulated system at different temperatures indicates that the density of NMP at the electrode interface exceeds that of water molecules (Figure 3k; Figure S20, Supporting Information). These findings suggest that NMP is adsorbed and enriched in aligning with the in‐situ infrared test results. The specific adsorption between electrodes and electrolytes is further evidenced by the contact angles between CNT and NMP. Compared to several common cosolvents, pure NMP exhibits the smallest contact angle, whereas the addition of water to NMP as a cosolvent resulted in the largest decrease in contact angle, indicating better wettability between NMP molecules and CNT electrodes, thus tending to preferentially adsorb NMP molecules (Figure 3l). Additionally, DFT calculations were utilized to investigate the specific adsorption between electrodes and different solvent molecules (Figure S21, Supporting Information). The NMP molecules possessed lower binding energy and were readily adsorbed on the electrode surface, indicating that OHEs containing NMP exhibit better compatibility with CNT electrodes.

In summary, the high output voltage and low‐temperature resistance of the flexible supercapacitor are attributed to the water deactivation induced by the synergistic effect of intermolecular interactions (solvation structure and H‐bonding) and IHP structural changes of the NMP in OHE‐4.5 (Scheme 1). As a cosolvent, NMP disrupts the H‐bonding network, inhibits the formation of ice crystals, participates in Li^+^ solvation, and maintains good ionic conductivity. Simultaneously, NMP molecules are more readily desolvated to be specifically adsorbed and enriched on the electrode surface, forming an H_2_O‐deficient IHP, further preventing water splitting.

### 2.4 Expanding the Output Voltage Limits of AFSC‐4.5 at Low Temperatures

**Figure 4:**
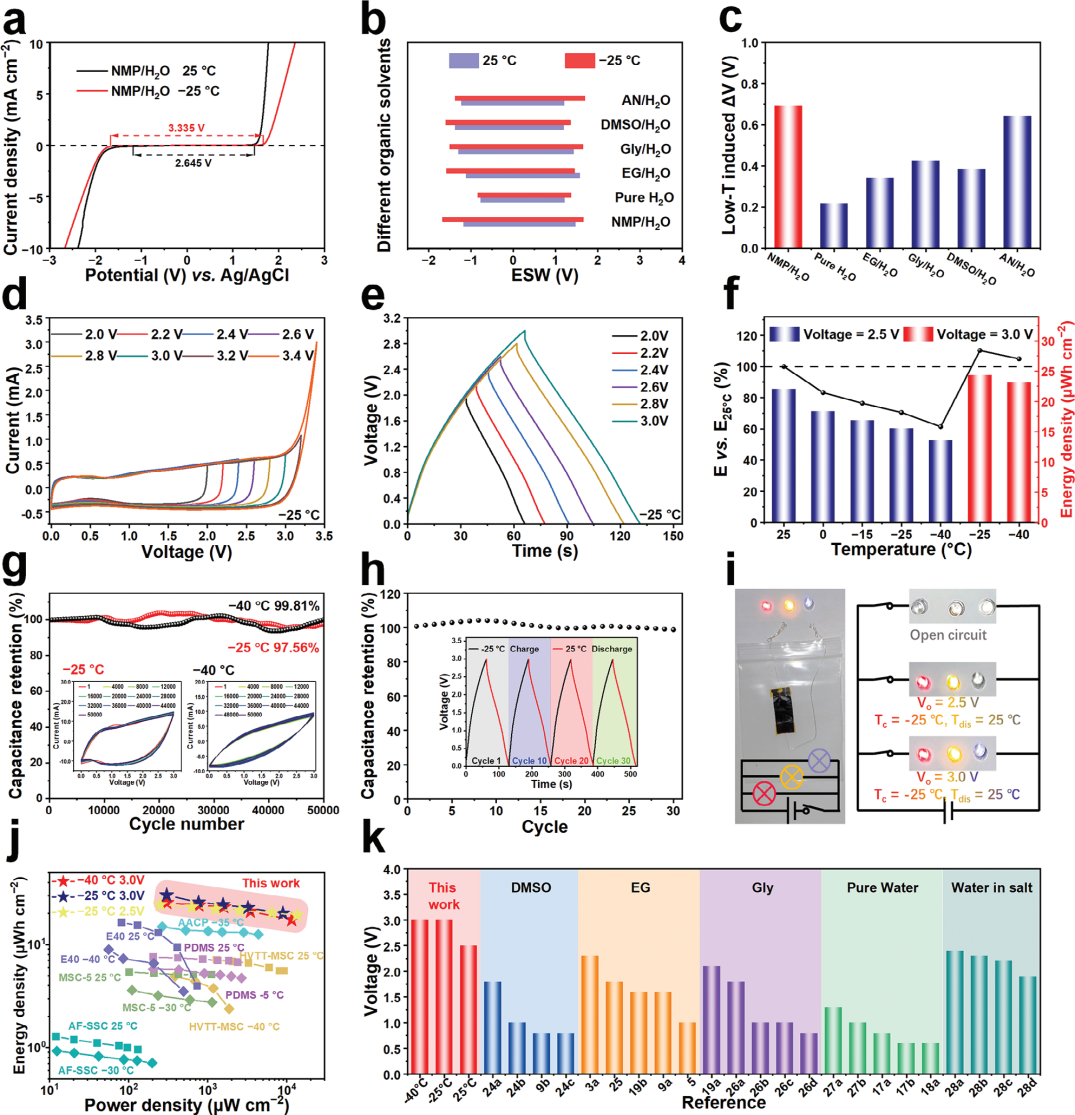
Low‐temperature‐induced enhancement of electrochemical properties of AFSC‐4.5. a) LSV curves of electrolytes containing NMP at different temperatures. b) ESW of electrolytes containing different types of additives at 25 °C and −25 °C. c) Changes in the ESW of different electrolytes. d) CV curves of AFSC‐4.5 in voltage ranges of 2.0–3.4 V at −25 °C. e) GCD curves of AFSC‐4.5 in voltage ranges of 2.0–3.0 V at −25 °C. f) Energy density retentions at 1.0 mA cm^−2^ of AFSC‐4.5. g) Cycling stability of AFSC‐4.5 under 0–3.0 V at −25 and −40 °C (The insets show the CV curves for stability at different temperatures). h) Capacitance retention of AFSC‐4.5 charging at −25 °C and discharging at 25 °C after 30 switches. i) The scheme of AFSC‐4.5 powers three LED lights connected in parallel (V_o_: output voltage, T_c_: charging temperature T_dis_: discharging temperature). j) Ragone plot of AFSC‐4.5 and other recently reported supercapacitors with aqueous gel electrolytes. k) Output voltage of AFSC‐4.5 at temperatures of −40, −25, and 25 °C, substantially exceeding previously reported supercapacitors using aqueous electrolytes at room temperature.

Based on the low‐temperature‐induced enhancement of intermolecular interactions, the increase in the content of NMP molecules in the IHP (Figure 3), and the reaction kinetics of water splitting, the reactivity of water in the OHE is further inhibited, which is expected to extend the voltage limit of supercapacitors at low temperatures. The impact of several common organic cosolvents on ESW at different temperatures was investigated through LSV (**Figure** **4**a; Figure S22, Supporting Information). Notably, low temperatures inhibit both hydrogen evolution and oxygen evolution reaction, with NMP as a cosolvent exhibiting the most significant voltage increase (including ΔV at Low‐T) compared to room temperature (Figure 4b,c). Consequently, the electrochemical performance of AFSC‐4.5 was further scrutinized over a broad temperature range (−40 to −25 °C) to demonstrate its capacity for broadening the voltage limit at the device level.

Illustrated in Figure 4d,e and Figure S23 (Supporting Information), at −25 °C and −40 °C, a notable polarization of the CV curve occurred at voltages exceeding 3.0 V, and the GCD curves from 2.0 to 3.0 V also exhibited stable charging and discharging behaviors. These findings indicate that AFSC‐4.5 can operate stably at 3.0 V, which is the highest output voltage achieved thus far by aqueous supercapacitors. Notably, the AFSC‐4.5 maintained nearly ideal electrical double‐layer capacitive behaviors from CV and GCD curves even under high scan rates and current densities at −25 and −40 °C under 3.0 V output voltage (Figure S24, Supporting Information). The higher output voltage and larger specific capacity endow the supercapacitor with higher energy density at low temperatures. In comparison to 25 °C (22.05 µWh cm^−2^), the AFSC‐4.5 with an output voltage of 3.0 V exhibits unprecedented energy densities of 110% (24.41 µWh cm^−2^) and 105% (23.16 µWh cm^−2^) at −25 and −40 °C, respectively (Figure 4f). Moreover, after 50 000 cycles for CV (97.56% and 99.81% at −25 and −40 °C, respectively) and GCD (92.09% at −25 °C) under 3.0 V output voltage, the capacitance retention results of AFSC‐4.5 further underscoring the reliability and broad applicability of AFSCs (Figure 4f; Figure S25, Supporting Information). Meanwhile, commercial active carbon (AC) material electrodes were employed to assemble the AC‐4.5 device. Following the same performance test, it demonstrated an ultrahigh output voltage of 2.5 V in the range of −40 to 25 °C (Figure S26, Supporting Information) and 3.0 V output voltage in the range of −40–−25 °C (Figure S27, Supporting Information).

The AFSC‐4.5 exhibits switchable output voltages at different temperatures. The device was charged to 3.0 V at low temperatures (−25 °C) and discharged at room temperature (25 °C). After 30 cycles of testing, the device's specific capacity remained stable (Figure 4h). To gain a more intuitive understanding of the practical impact of output voltage, a single AFSC‐4.5 was utilized to illuminate parallel red, yellow, and blue LED lights (Figure 4i). A feasible approach is to charge it to 3.0 V at −25 °C and discharge it at room temperature to illuminate three LEDs simultaneously. The switchable output voltage not only provides a higher energy density for the capacitor but also broadens the practical usage scenarios of the device. AFSC‐4.5 exhibits outstanding energy density at different temperatures, particularly at low temperatures, surpassing some previously reported flexible supercapacitors. When compared to other aqueous supercapacitors, the electrochemical performance of AFSC‐4.5 at low temperatures was found to be superior in terms of energy density, power density, and rate capability (Figure 4j; Table S2, Supporting Information).^[^ ^3^, ^18^, ^19^, ^23^ ^]^ Concurrently, the output voltages of AFSC‐4.5 at temperatures of −40, −25, and 25 °C substantially exceeded those of previously reported symmetrical supercapacitors utilizing aqueous electrolytes at room temperature (including electrolytes with cosolvent dimethyl sulfoxide (DMSO),^[^ ^9^, ^24^ ^]^ ethylene glycol (EG),^[^ ^3^, ^5^, ^9^, ^19^, ^25^ ^]^ glycerol (Gly),^[^ ^19^, ^26^ ^]^ and pure water,^[^ ^17^, ^18^, ^27^ ^]^ and even surpassed most WIS electrolytes^[^ ^28^ ^]^ (Figure 4k; Table S3, Supporting Information). The electrochemical data of AFSC‐4.5 at different temperatures are summarized in Table S4 (Supporting Information), including output voltage, specific capacity, energy density, and power density. To the best of our knowledge, AFSC‐4.5 is the first carbon‐based device to offer such high output voltage and area energy density across the wide temperature range of −40 to 25 °C.

## 3 Conclusion

This study aimed to enhance the output voltage limit of AFSCs by incorporating water‐deactivation OHEs. Through theoretical calculations and experimental analyses, we have elucidated the critical role of the cosolvent NMP in regulating water deactivation in terms of intermolecular interactions (solvation structure and H‐bonding) and IHP regulation of OHEs, which increases the output voltage of AFSCs. OHE‐4.5 effectively suppresses the water decomposition reaction, allowing AFSCs to operate at a record voltage of 2.5 V across temperatures ranging from −40 to 25 °C and 3.0 V within the range of −40–−25 °C, surpassing the performance of all reported symmetric supercapacitors with aqueous electrolytes. Moreover, the AFSCs demonstrate remarkable environmental adaptability and stability, maintaining 97.0% and 80.5% of the initial capacitance at 0−2.5 V after 50 000 cycles at −40 °C or 20 000 cycles at 25 °C, respectively, and retaining >95% of the capacitance even after 50 000 cycles at 0–3.0 V (−25 and −40 °C). With an output voltage of 3.0 V, AFSC‐4.5 achieves unprecedented energy densities of 110% (24.41 µWh cm^−2^) and 105% (23.16 µWh cm^−2^) at −25 and −40 °C, respectively, compared to 25 °C (22.05 µWh cm^−2^). Notably, the ability to switch the output voltage at different temperatures offers the potential for electronic devices to operate effectively in low‐temperature environments. This study opens up exciting possibilities for designing advanced electrolytes that enable AFSCs to achieve high output voltages across a wide operating temperature range.

## 4 Experimental Section

### Materials

Organic solvents include N‐methylpyrrolidone (NMP, 98%), dimethyl sulfoxide (DMSO, 98%), ethylene glycol (EG, 98%), glycerol (Gly, 99%), acetonitrile (AN, 98%), and polytetrafluoroethylene preparation (PTFE, 60 wt% in water) were purchased from Macklin. polyvinyl alcohol (PVA, 130 000 MW) was purchased from Sigma‐Aldrich. Lithium trifluoromethanesulfonate (LiOTf, 99.5%) was purchased from Aladdin. Carbon nanotube paper (CNT), active carbon (AC), and conductive carbon black were purchased from XFNANO.

### Preparation of PVA Organogel and OHEs

A 2.0 g PVA powder was first dissolved in 18.0 g NMP solution at 110 °C with stirring. Subsequently, the obtained PVA (10 wt%) homogeneous solution was injected into the mold and refrigerated at −25 °C for 6 h to obtain PVA organogel. Then, six solutions were configured with LiOTf concentrations of 1, 2, 3, 4, 4.5, and 5 m (the solvent mixture used had a mole fraction of NMP of 4% and a mole fraction of H_2_O of 96%) and were used to soak PVA organogels to obtain OHEs (Corresponding to OHE‐1, OHE‐2, OHE‐3, OHE‐4, OHE‐4.5 and OHE‐5 according to salt concentration). The OHE was named according to the salt concentration, namely the OHE obtained by immersing in a solution containing 4% NMP and 4.5 m LiOTf for 24 h is named OHE‐4.5.

### Preparation of Liquid Electrolytes

The composition of liquid electrolytes (LEs) is consistent with the soaking solutions mentioned above and after replacing NMP with other organic solvents of the same amount of substance, a series of liquid electrolytes were obtained for testing the ESW at different temperatures.

### Preparation of CNT Electrodes and Assembly of Supercapacitors

The preparation of commercial AC electrodes is achieved by ultrasonically dispersing activated carbon, conductive carbon black, and PTFE in a 7:2:1 mass ratio in an ethanol solution, then OHE‐4.5 was assembled in a sandwich structure to form a symmetrical AC‐4.5.

### Preparation of AC Electrodes and Assembly of Supercapacitors

The carbon nanotube paper was cut into measuring 1.0 cm × 2.0 cm and Ag wires were fixed on the pieces by Ag glue. After half an hour of drying the silver glue in a blast oven, a conductive CNT electrode is obtained. Subsequently, two prepared CNT electrodes and OHE‐4.5 were assembled in a sandwich structure to form a symmetrical AFSC‐4.5. The assembled AFSC‐4.5 is simply packaged with a tape for electrochemical performance testing.

### Characterizations

The intermolecular interaction of samples was investigated using Raman spectroscopy with a HORIBA XploraPlus Raman Microscope. Fourier transform infrared (FTIR) spectroscopy (Thermoscientific Nicolet iS10) within the range of 400–4000 cm^−1^. The in‐situ FTIR testing device uses symmetrical carbon cloth electrodes and applies voltage through the CHI 660e electrochemistry workstation.

Differential scanning calorimetry (DSC) was performed on Discovery DSC 250. The temperature was ranging from −90 to 50 °C, at a rate of 10 °C min^−1^. Rheological tests of the electrolyte samples (1.5 cm in diameter and 0.2 cm in thickness) were carried out by using an Anton Paar model MCR‐301 rheometer (Austria) at the temperature arranged from −50 to 50 °C. The storage moduli (G’) and loss moduli (G’’) of the electrolytes were measured on a frequency weep of 10 rad s^−1^ at a constant strain of 0.1%. Tensile tests were carried out using a versatile testing machine (Shimadzu AGS‐X, Japan) at 25 and −25 °C, respectively. The OHE‐4.5 was cut into a dumbbell shape and tested with a strain rate of 50 mm min^−1^.

Test the contact angle of different solvents on CNT electrodes using a contact angle measuring instrument (SDC‐350). A 2.0 µL droplet was dropped on the CNT surface and the contact angles between different solutions and CNT were obtained through fitting calculations.

### Electrochemical Performance Testing

All electrochemical performance measurements were conducted through the CHI 660e electrochemical workstation. In a three‐electrode system, linear sweep voltammetry (LSV) was used to study the electrochemical stability window (ESW) of different salt solutions at a scanning rate of 10 mV s^−1^, and glassy carbon, Pt, and Ag/AgCl as with the working, counter, and reference electrode, respectively. The sandwich structure assembled by two Pt electrodes and gel electrolyte was subjected to electrochemical impedance spectroscopy (EIS, frequency range 0.1–10^5^ Hz), and the ionic conductivity (mS cm^−1^) at different temperatures was calculated by Equation (1):

(1) $$\sigma =\frac{L}{R\times S}$$

where L, R, and S are the thickness, the resistance, and the area of electrolytes, respectively.

The energy storage performance of assembled supercapacitors was studied by testing their galvanostatic charge–discharge (GCD) curves and cyclic voltammetry curves in the temperature range of −40–25 °C. The specific capacity of supercapacitors (C_a_ mF cm^−2^ or C_m_ F g^−1^) was calculated using Equations (2) and (3):

(2) $$C_{a}=\frac{I\times \Delta t}{V\times A}$$

(3) $$C_{m}=\frac{I\times \Delta t}{V\times m}$$

Calculate the energy density (E, µWh cm^−2^) and power density (P, µW cm^−2^) of AFSC according to Equations (4) and (5) and evaluate supercapacitors in terms of energy storage and output:

(4) $$E=\frac{C\times V^{2}}{7200}$$

(5) $$P=\frac{3600E}{\Delta t}$$

where Δt, I, U, A, and m are the discharging time, the discharge current (mA), the voltage minus the IR drop (V), the area of the CNT electrode (cm^2^), and the mass of activated carbon in AC electrode, respectively.

When the testing temperature involves a low‐temperature environment, in order to prevent the Ag/AgCl reference electrode from freezing and not working properly at low temperatures, a KCl solution prepared with water/NMP mixed solvent was chosen to replace the original solution of the reference electrode to ensure the stability of the reference electrode potential during the testing process. According to the Arrhenius equation, R_ct_ has strong ties to temperature characteristics as follows: The desolvation‐free energy corresponding to the electrolyte was calculated and fitted through the impedance testing of a three‐electrode system at different temperatures. The relationship Equation is:

(6) $$\frac{1}{R_{ct}}=A_{0}e^{-\frac{E_{a}}{RT}}$$

where, R_ct_, A_0_, E_a_, R, and T are the transfer resistance (Ω), exponential factor, desolvation free energy (kJ mol^−1^), gas constant, and temperature (K), respectively.

### Theoretical Calculations

All of the DFT calculations were using the Orca program package.^[^ ^29^ ^]^ The binding energies of the Li^+^‐solvent and electrode‐solvent complexes were calculated after structural optimizations. The binding energy was calculated as:

(7) $$\mathrm{Ebin}=E\left(\mathrm{AB}\right)-\left[E\left(A\right)+E\left(B\right)\right]$$

Additionally, the electronic structure of Li^+^−4H_2_O and Li^+^−NMP−3H_2_O clusters are calculated, and the ESP of the clusters was analyzed by Multiwfn program version 3.8‐dev.^[^ ^30^ ^]^ The molecular dynamics (MD) simulations were performed using the GROMACS package^[^ ^31^ ^]^ and the computational model was randomly assembled through the PACKMOL package.^[^ ^32^ ^]^ The GAFF force field parameters were used to describe bonded and nonbonded interactions for studied molecules.^[^ ^33^ ^]^ Moreover, the electrostatic potential and MD model are generated and rendered using the visualization software package VMD.^[^ ^34^ ^]^

## Conflict of Interest

The authors declare no conflict of interest.

## Supporting information



Supporting Information

## Data Availability

The data that support the findings of this study are available from the corresponding author upon reasonable request.
