# Supplementary material for: High Output Voltage Aqueous Supercapacitors by Water Deactivated Electrolyte over Wide Temperature Range
Source: Adv Sci (Weinh). 2025 Feb 18;12(14):2500385. doi: 10.1002/advs.202500385 (PMC11984914; doi:10.1002/advs.202500385)
Supplement: Supplementary file 1 — Supporting Information [file ADVS-12-2500385-s001.docx]

Supporting Information

High output voltage aqueous supercapacitors by water deactivated electrolyte over wide temperature range

Hongji Wang, Wenpeng Liu, Jin Huang, Tianliang Xiao, Wenwei Lei,* Faming Gao,* and Mingjie Liu*

**This PDF file includes:**

**Figures S1 to S27**

**Tables S1 to S4**


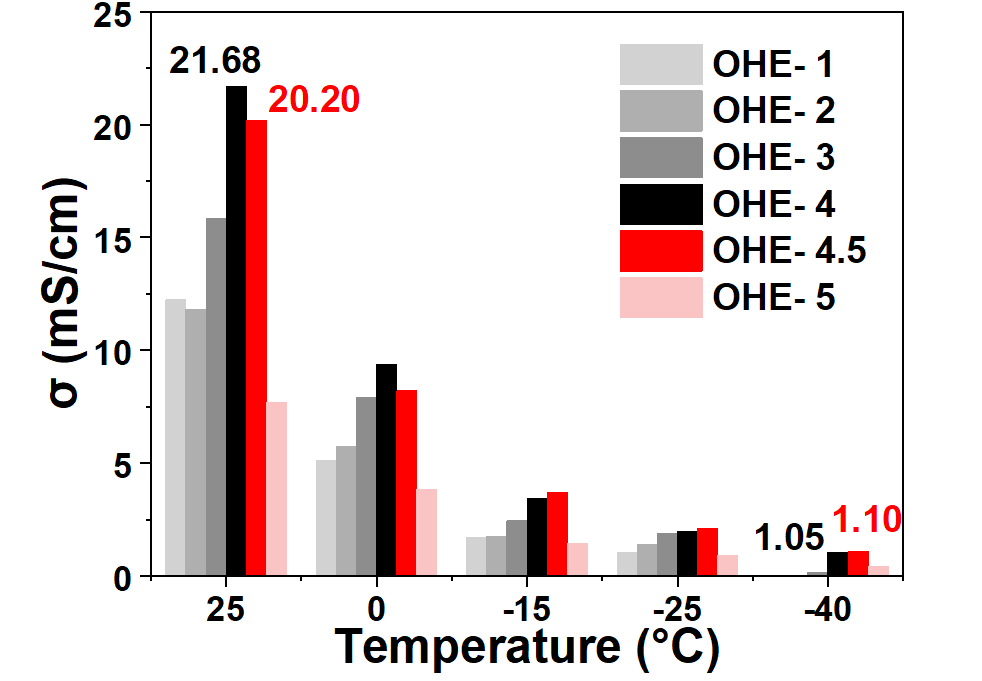


**Figure S1.** Ionic conductivity of OHEs under the temperature.


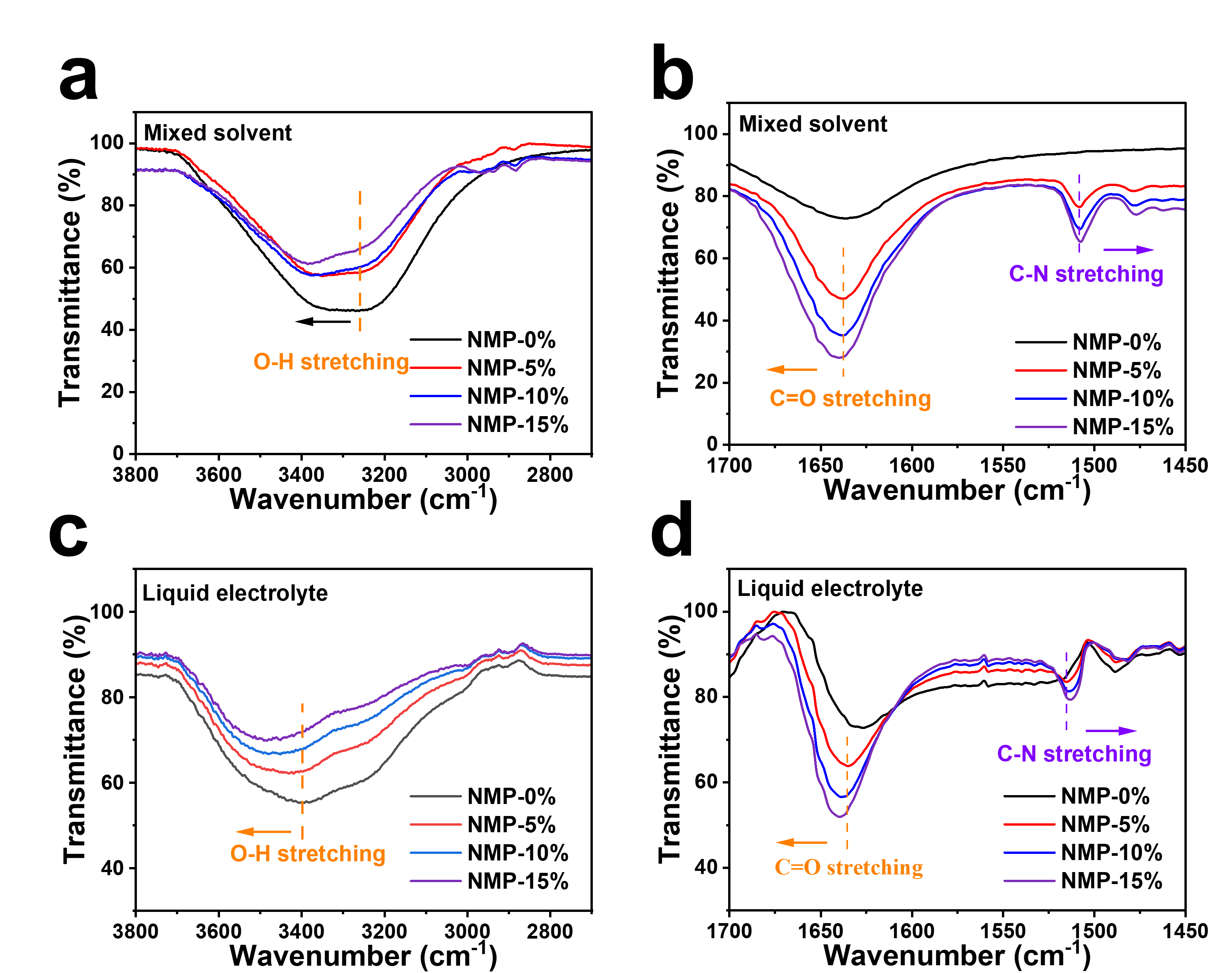


**Figure S2.** FTIR spectra investigate the hydrogen bonding interactions in water/NMP solutions and LEs with different NMP contents. (a, b) The FTIR vibration peaks of O−H belong to H_2_O, C=O and C−N belong to NMP in NMP/H_2_O solutions. (c, d) The FT-IR vibration peaks of O−H belong to H_2_O, C=O and C-N belong to NMP in liquid electrolytes.


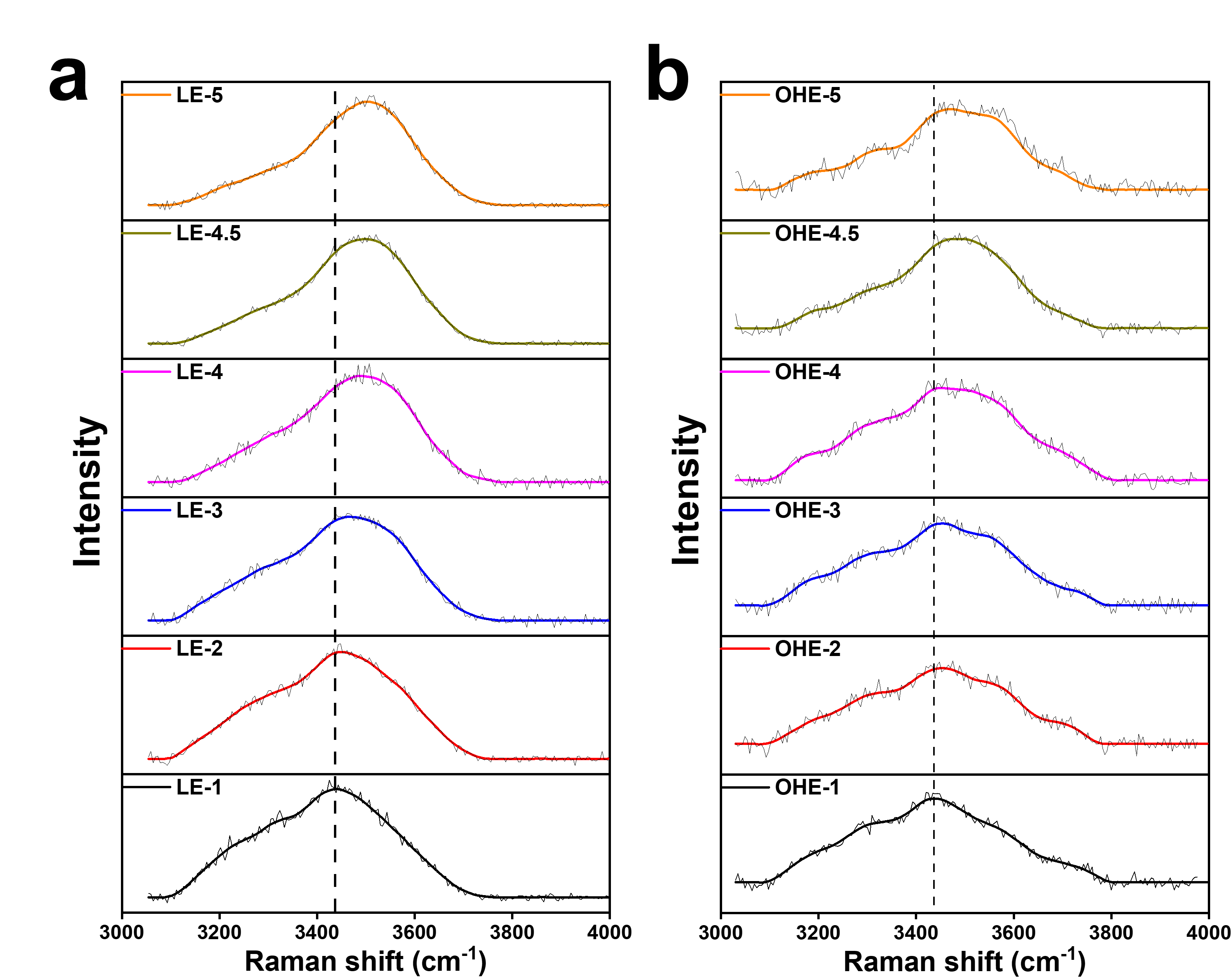


**Figure S3.** Raman spectra investigate the hydrogen bond strength among the water molecules in LEs and OHEs with different LiOTf concentrations. (a, b) The Raman spectra of LEs and OHEs with different LiOTf concentrations and the electrolytes about O−H stretching vibration peak shifts to higher wavenumbers as the salt concentration increases.

# Raman spectra and peaks fitting of OHEs

As shown in **Figure S4,** the Raman spectra in the 3000–4000 cm^−1^ region of OHEs were analyzed by peak fitting. In the OHEs, water molecules roughly exhibit four types of hydrogen bonding interactions: SHW (strongly hydrogen-bonded water), MHW (medium hydrogen-bonded water), WHW (weak hydrogen-bonded water), and PHW (hydrogen bonding interactions by PVA chains with water) that also have O−H further complicate the hydrogen bonds within the system. The O−H peaks of OHEs were deconvolved to better investigate the interactions involving water molecules in OHE.


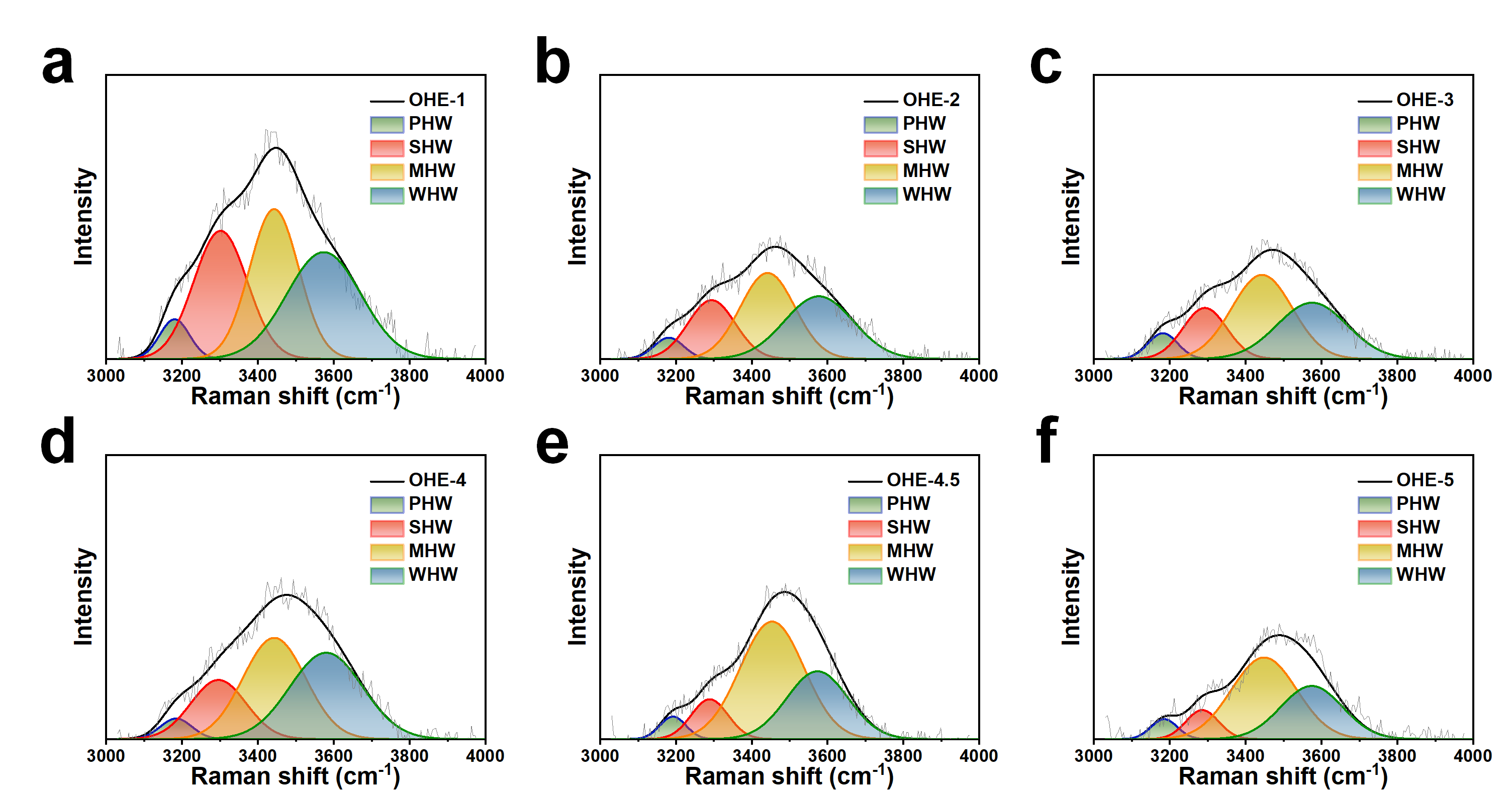


**Figure S4.** Raman spectra and peaks fitting of OHEs with different LiOTf concentrations.


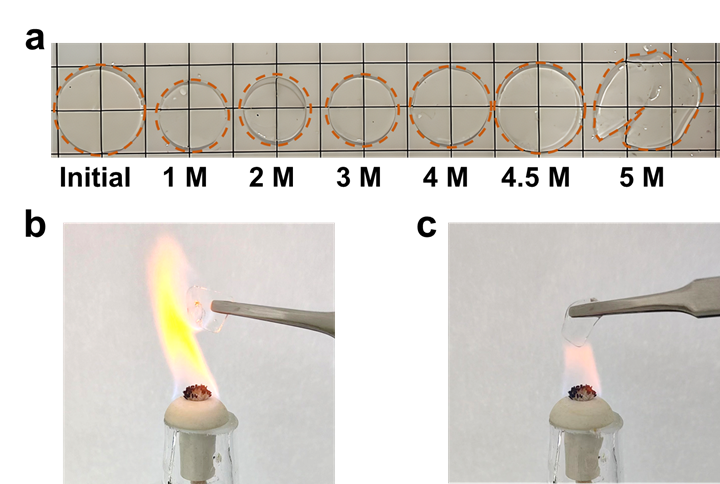


**Figure S5.** Pictures of OHEs with different LiOTf concentrations and ignition experiments of PVA organogel and OHE-4.5. (a) PVA organogel after immersion in LEs with different concentrations for 24 h, OHE-4.5 can still maintain its original state. (b, c) In the ignition experiment PVA organogel is more flammable than OHE-4.5.

# The ESW of OHEs with different LiOTf concentrations

In **Figure S6**, the LSV curves (10 mV s^−1^) of different OHEs in the Swagelok system (CNT as work electrode, Ti as counter electrode, and anti-freezing Ag/AgCl as reference electrode), as the LiOTf concentrations increase the ESW of OHEs have expended. Detailed test results show that OHE-4.5 has the widest ESW when potentials are read at a current density of ±0.1 mA cm^−2^ (1.14 V for OHE-1, 2.34 V for OHE-2, 2.44 V for OHE-3, 2.88 V for OHE-4 and 3.17 V for OHE-4.5).


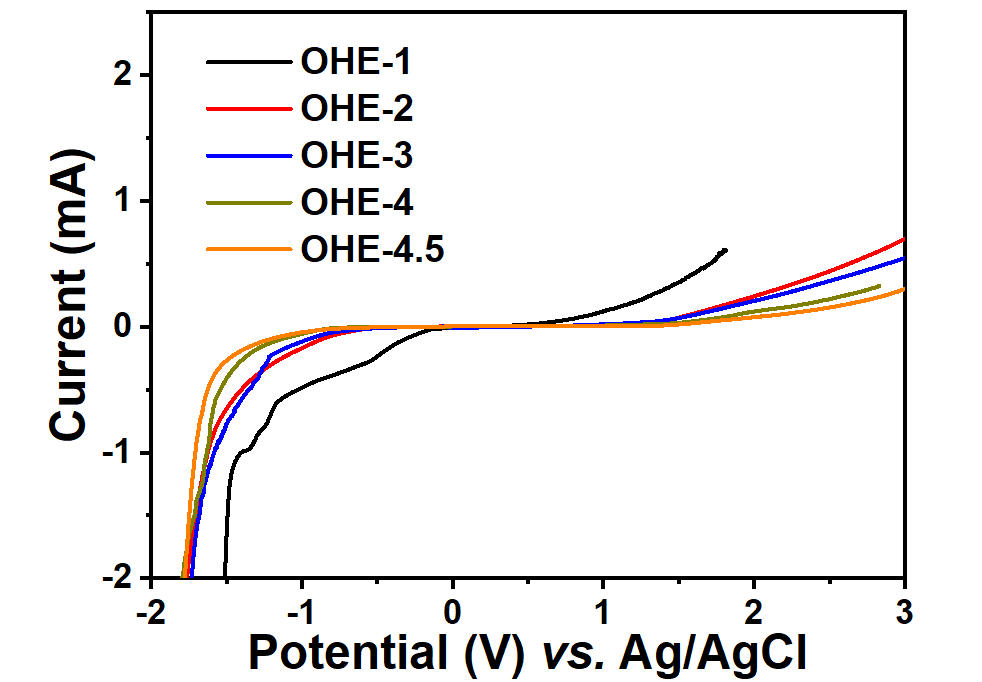


**Figure S6.** LSV tests of different OHEs by the Swagelok system. The scan rates of LSV curves of OHEs with different LiOTf concentrations are 10 mV s^−1^.


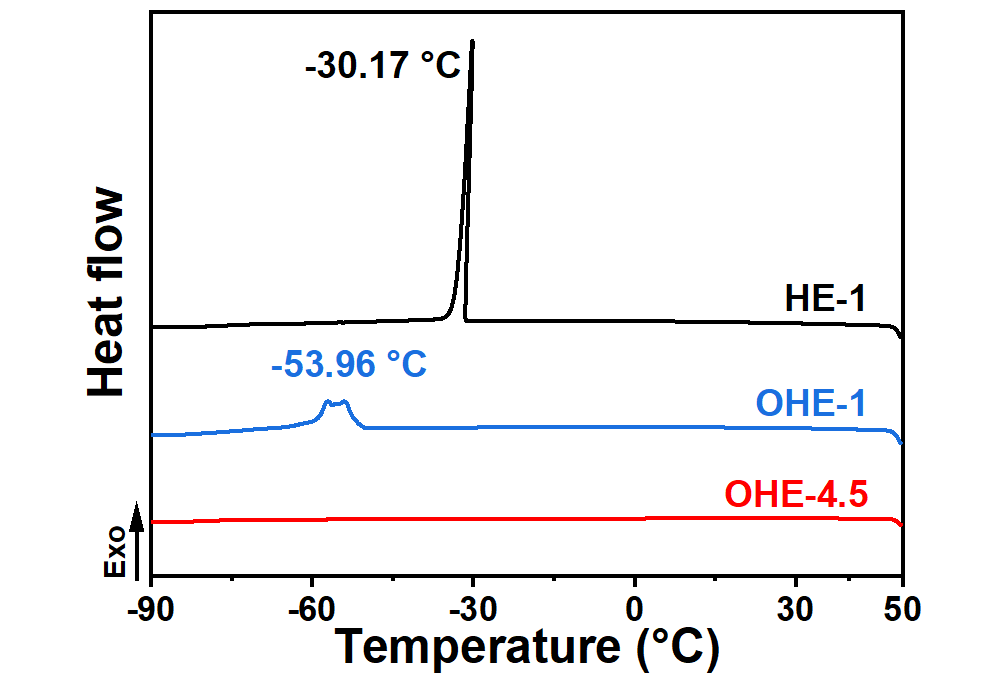


**Figure S7.** DSC curves of gel electrolytes during cooling, OHE-4.5 and OHE-1 without exothermic peaks in the range from 50 to −90 °C, while the water molecules in HE-1 and OHE-1 froze near −30.17 and −53.96 °C, respectively.

# Electrochemical stability of different OHE

To compare the electrochemical stability of the devices assembled with different electrolytes and CNT electrodes, the CV curves were tested (10 mV s^−1^) in the voltage range of 0–2.5 V. The OHE-4.5 has a weaker water decomposition current in OHEs (**Figure S8a**), and at the same LiOTf concentration, the HE-4.5 without NMP has a more pronounced decomposition current than OHE-4.5 (**Figure S8b**).


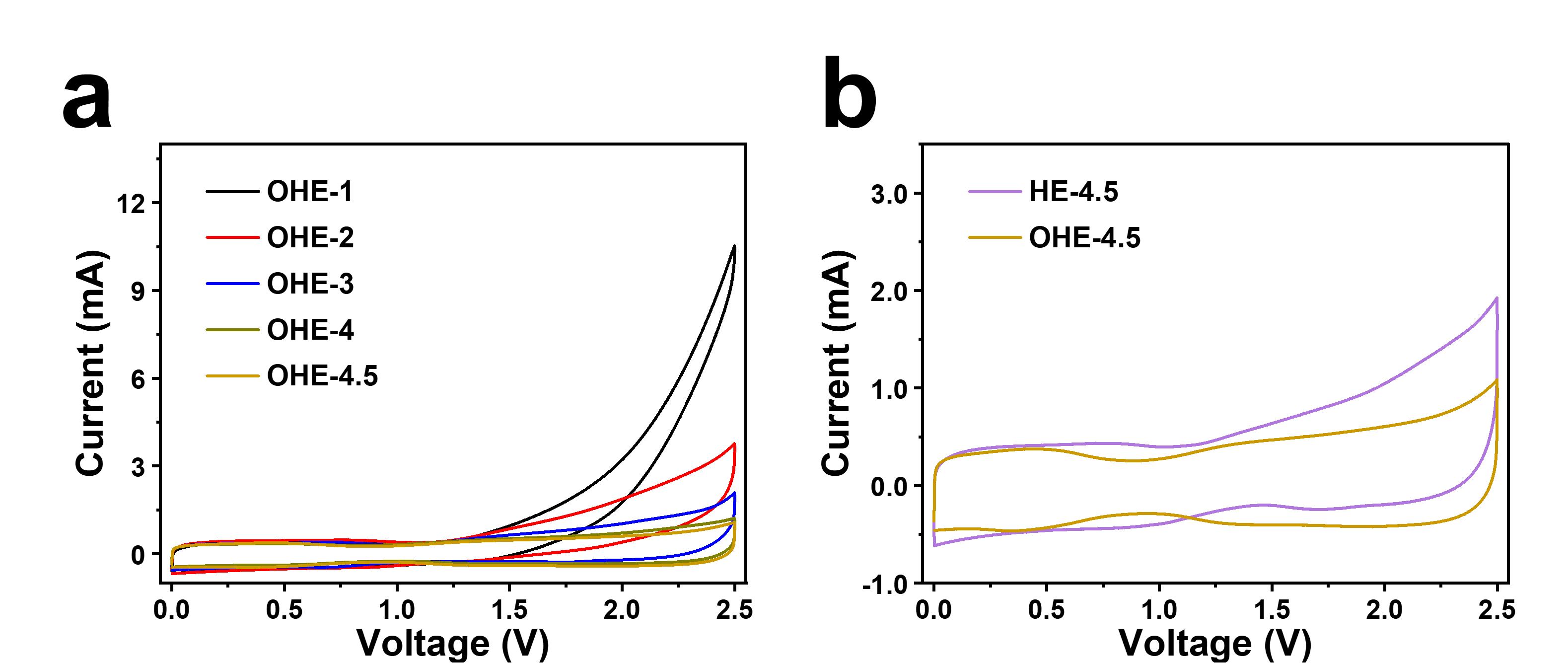


**Figure S8.** Output voltage of AFSCs assembled by different OHEs. (a) CV curves of OHEs with different LiOTf concentrations at 0–2.5 V. (b) CV curves of OHE-4.5 and HE-4.5 at 0–2.5 V.


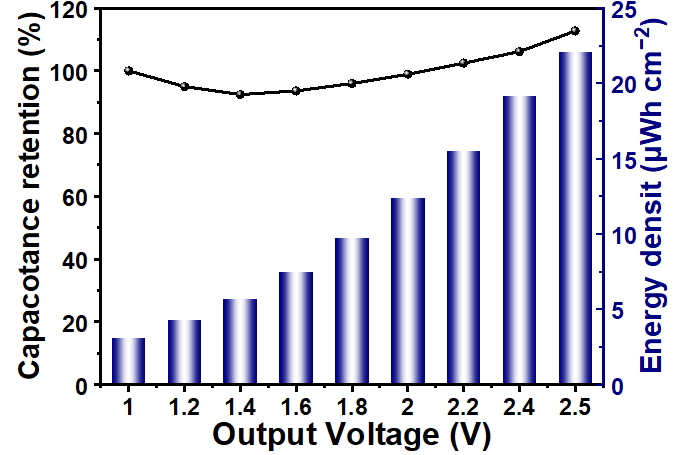


**Figure S9.** The energy density of AFSC-4.5 at different output voltages.


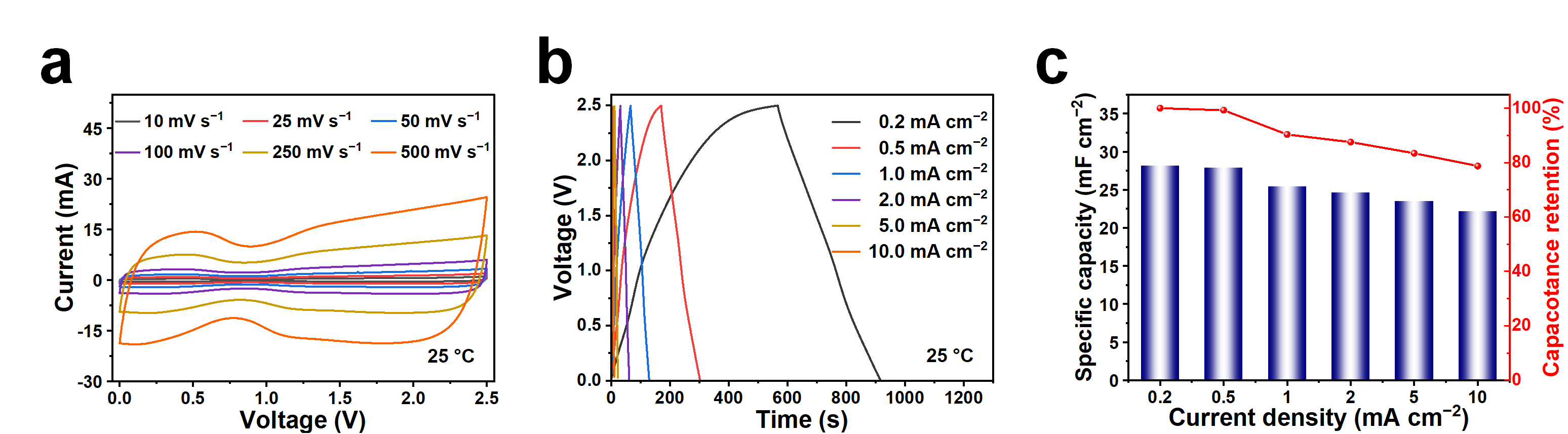


**Figure S10.** Electrochemical performance of AFSC-4.5 at 25 °C under 2.5 V output voltage. (a) CV curves at different scan rates. (b) The GCD curves at different current densities. (c) The rate performance of AFSC-4.5. AFSC-4.5 at a current density of 10.0 mA cm^−2^ can still have a capacity retention rate of 78.75%.


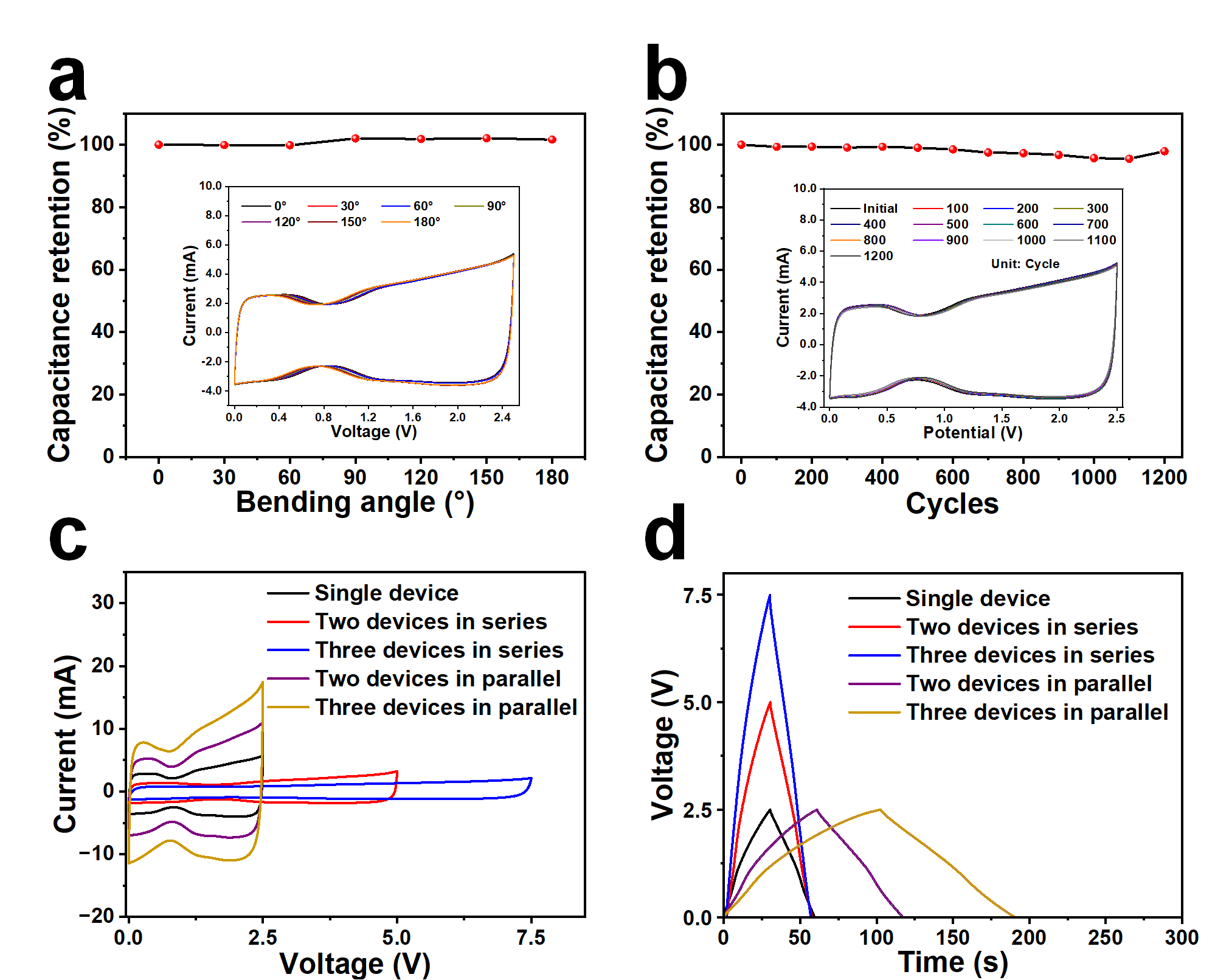


**Figure S11.** The mechanical and integrated performance of AFSC-4.5. (a) The capacity retention of AFSC-4.5 at different bending angles, inset is CV curves of AFSC-4.5 at different bending angles at 50 mV s^−1^. (b) The capacitance retention of AFSC-4.5 at 50 mV s^−1^ after 1200 bends. Inset is CV curves of AFSC-4.5 at different numbers of bending from 0 to 180 °. (c, d) Electrochemical performance of three AFSC-4.5 with series/parallel connections.

# The performance of AFSC-4.5 at low temperature

The electrochemical performance test of AFSC-4.5 at low temperatures shows the excellent low-temperature resistance of OHE-4.5. CV tests at different scan rates and GVD at different current densities were conducted on AFSC-4.5 at 0 °C (**Figures S12a-c**), −15 °C (**Figures S12d-f**), −25**°**C (**Figures S12g-i**), and −40 °C (**Figures S12j-l**). The CV curve of AFSC-4.5 can maintain a nearly rectangular surrounding area and a symmetrical triangle in a wide temperature range, which indicates that AFSC-4.5 has excellent low-temperature resistance performance. Especially at −40 °C, AFSC-4.5 still has a 15.61 mF cm^−2^ specific capacity and outputs an energy density of 13.55 μWh cm^−2^ at the power density of 1374.05 μW cm^−2^. More AFSC-4.5 performance data can be found in Supplementary Table S4.


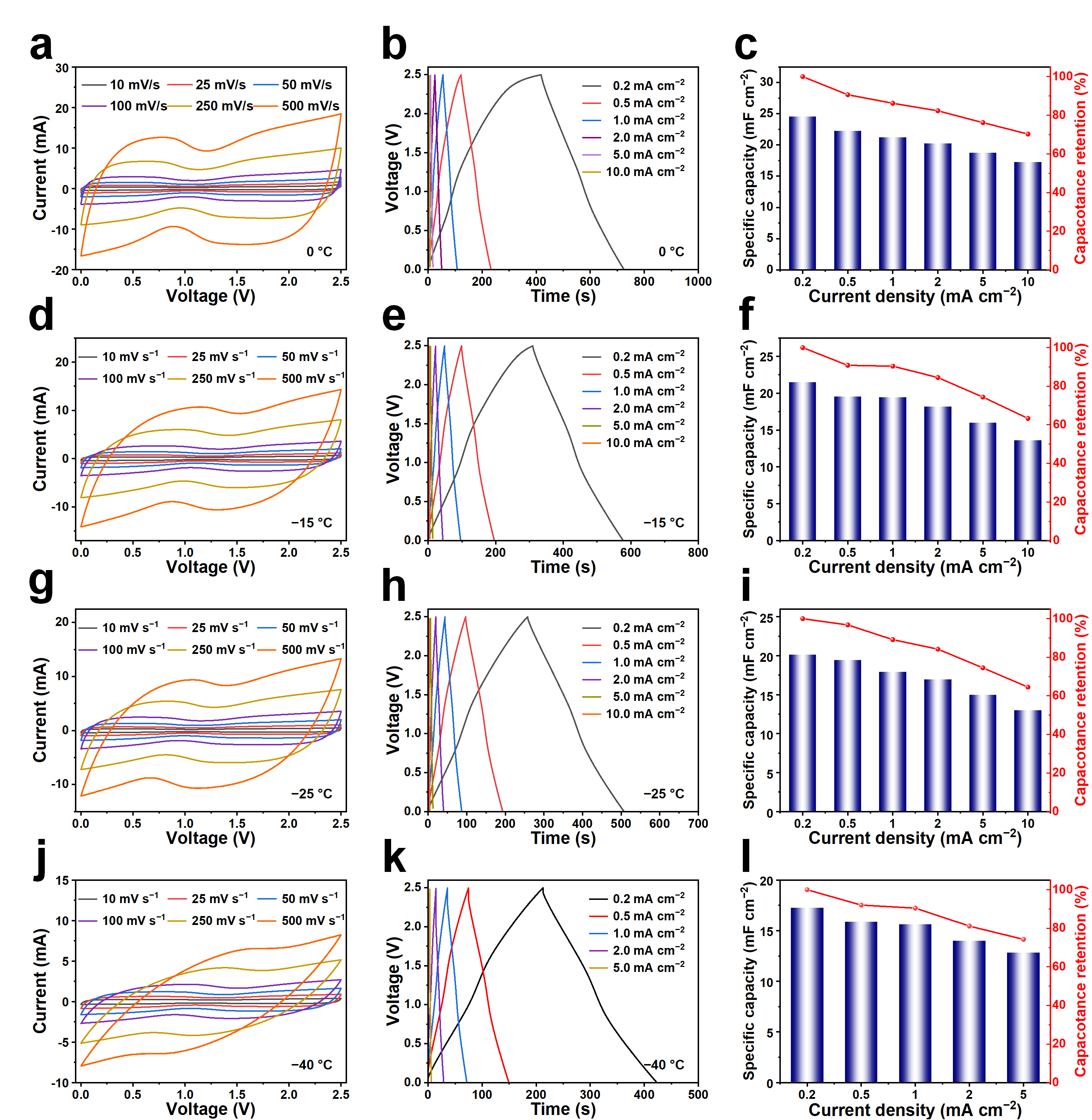


**Figure S12.** Performance of AFSC-4.5 at −40–0 °C. The electrochemical performance of AFSC-4.5 under 2.5 V output voltage at (a, b, c) 0 °C, (d, e, f) −15 °C, (g, h, i) −25 °C, (j, k, l) −40 °C, respectively.


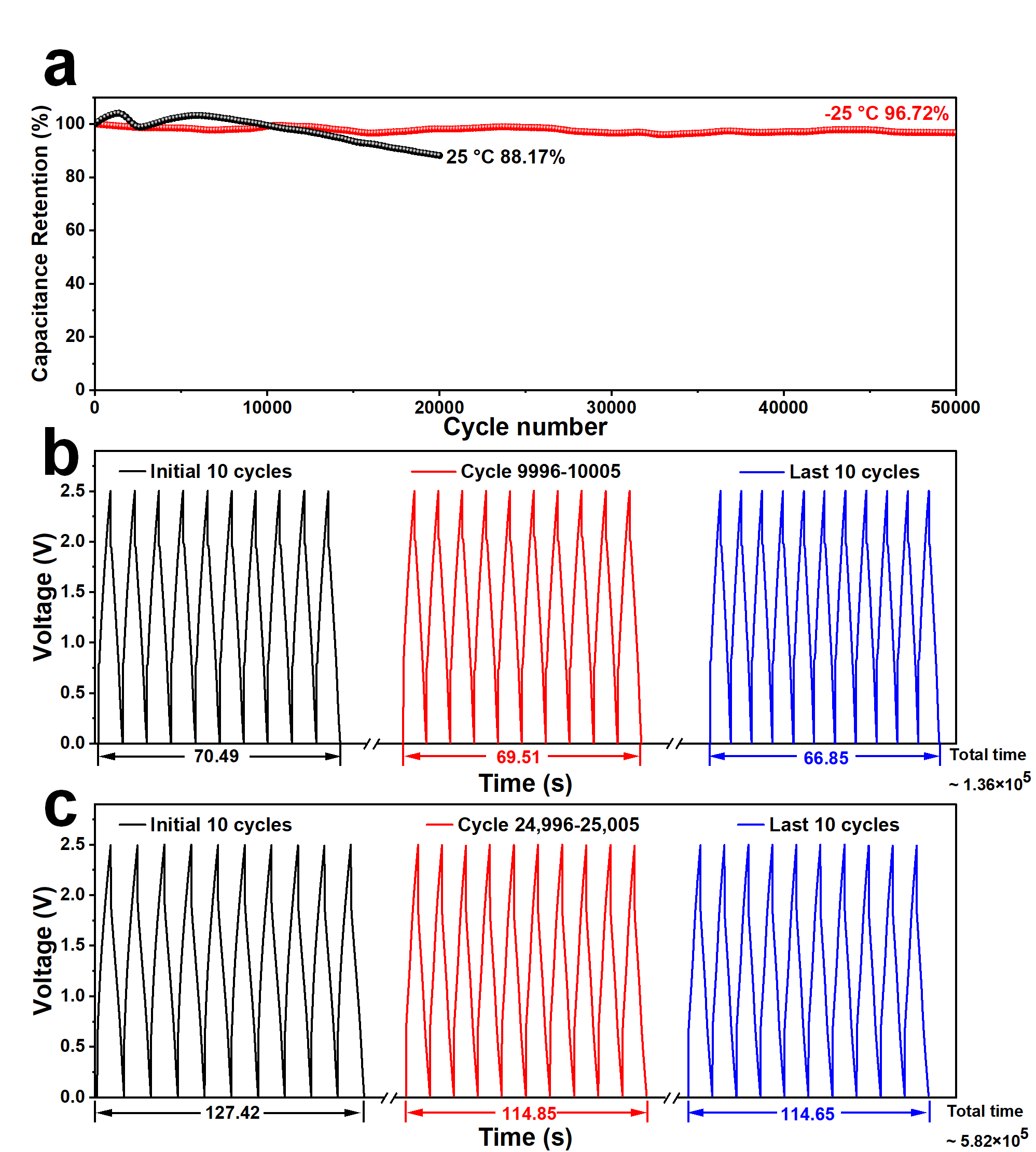


**Figure S13.** Stability of AFSC-4.5 at 2.5 V tested by GCD. (a) Stability of the AFSC-4.5 under 2.5 V voltage. Detailed data from the GCD stability test at (b) 25 (10.0 mA cm^−2^) and (c) −25 °C (5.0 mA cm^−2^).


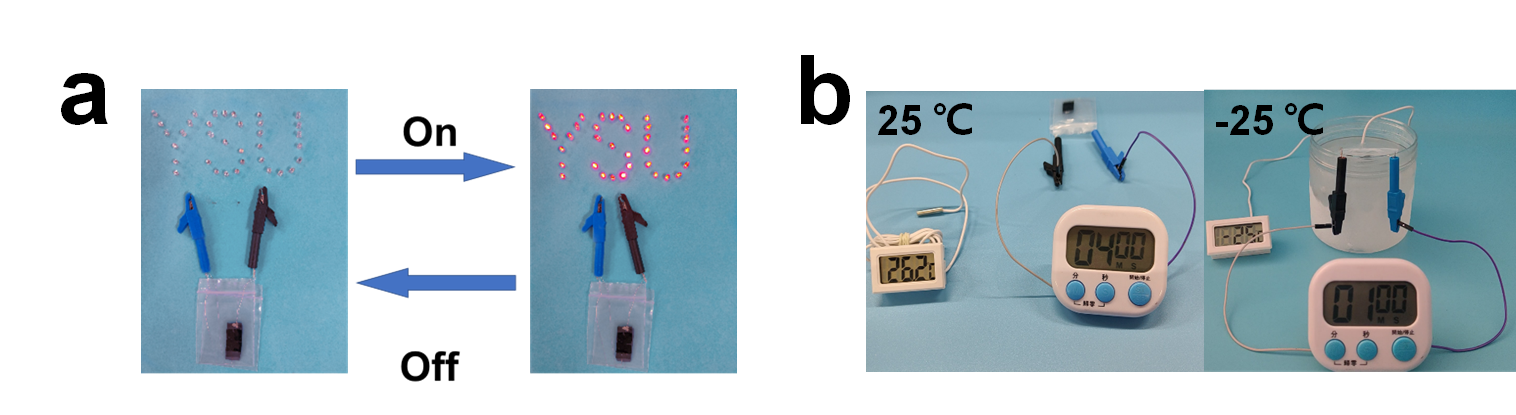


**Figure S14.** AFSC-4.5 power the electronics. (a) AFSC-4.5 lights up the ‘YSU’ LEDs. (b) AFSC-4.5 powering a timer at 25 and −25 °C, respectively.

# MD simulations and solvation structures within the model

Two models with the random distribution of internal molecules form a solvated structure containing ions and solvent molecules after MD simulation. In the pure H_2_O electrolyte model, there is only one solvent of water molecules in the solvation sheath (**Figure S15a**). However, after adding NMP, there are two solvents of water molecules and NMP molecules in the solvation sheath (**Figure S15b**).


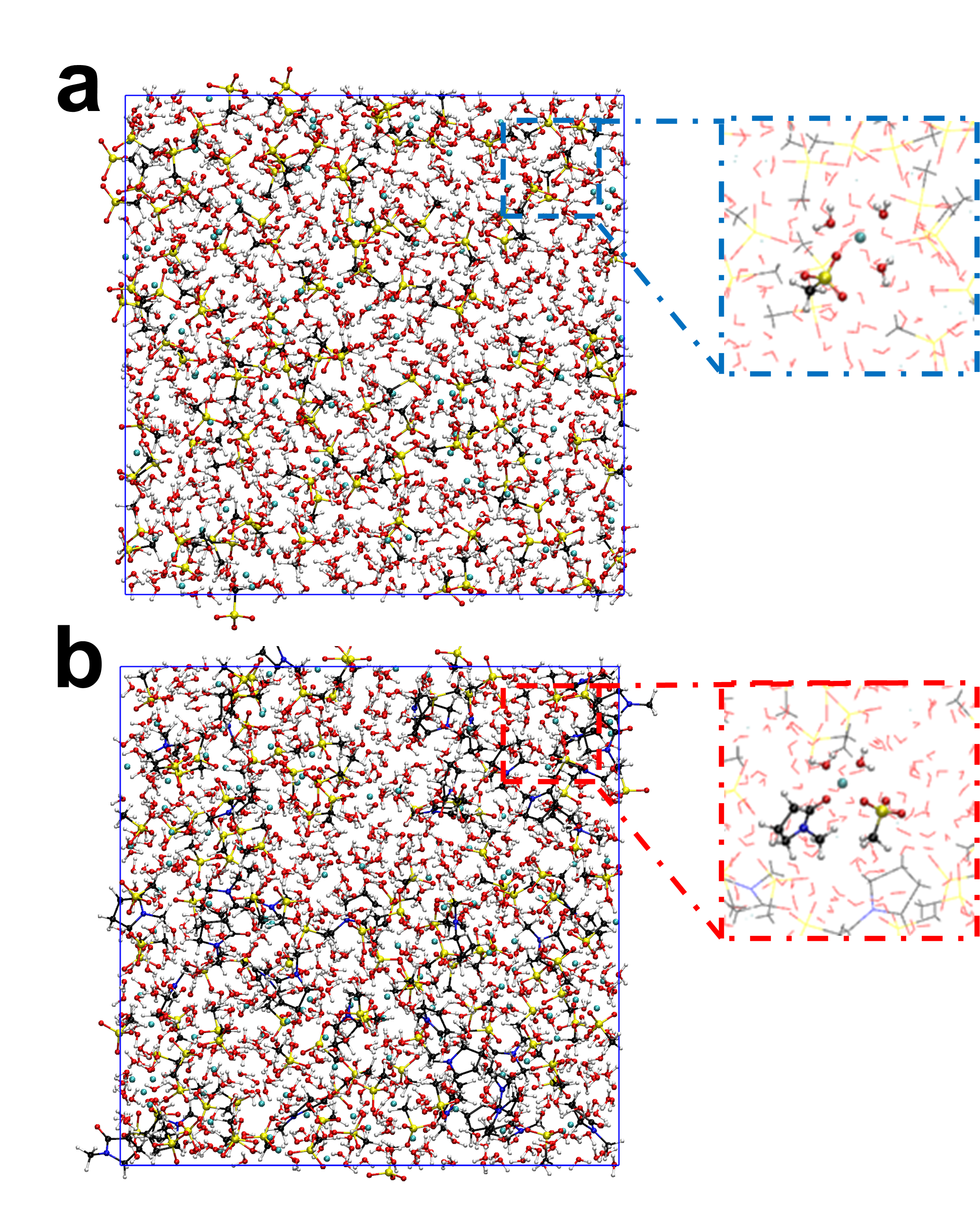


**Figure S15.** MD simulation model and partially solvated structures. (a) MD model with only water molecules for research HE. (b) MD model with NMP for research OHE.

As the simulation temperature changes, same simulation models express different solvation structures at −25 °C and 25 °C. The change trend of coordination number of solvent molecules around Li^+^ is similar, but opposite to the change of OTf^−^. Li^+^ is more inclined to combine with solvent molecules, weakening the interaction between anions and cations, which is conducive to better low-temperature resistance (**Figure S16**).


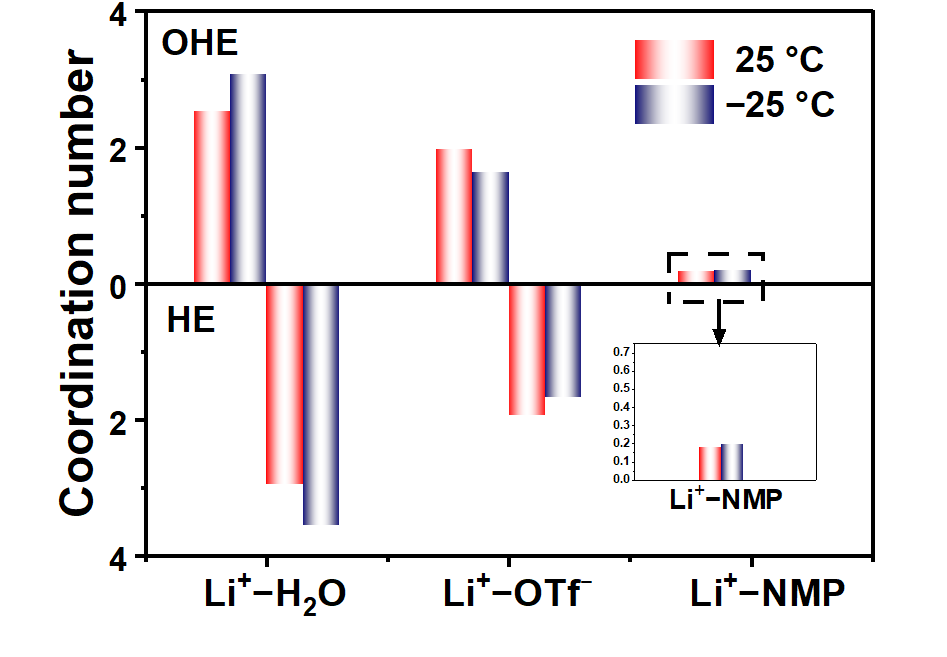


**Figure S16.** Temperature-dependent changes in the coordination number of Li^+^−solvents or Li^+^−OTf^−^ in HE and OHE.

# The influence of NMP content on electrolytes in impedance testing

As shown in **Figure S17,** EIS tests at different temperature were analyzing the effect of NMP content on the desolvation free energy of electrolytes. The value of R_ct_ is obtained from the corresponding EIS curve, and then the free energy of desolvation is fitted. A three-electrode system with a glassy carbon electrode as the working electrode, a platinum electrode as the counter electrode, and an anti-freezing Ag/AgCl electrode as the reference electrode. The anti-freezing Ag/AgCl electrode is a KCl solution prepared using NMP-H_2_O mixed solvent to replace the original KCl aqueous solution, in order to prevent the influence of low temperature on the reference electrode.


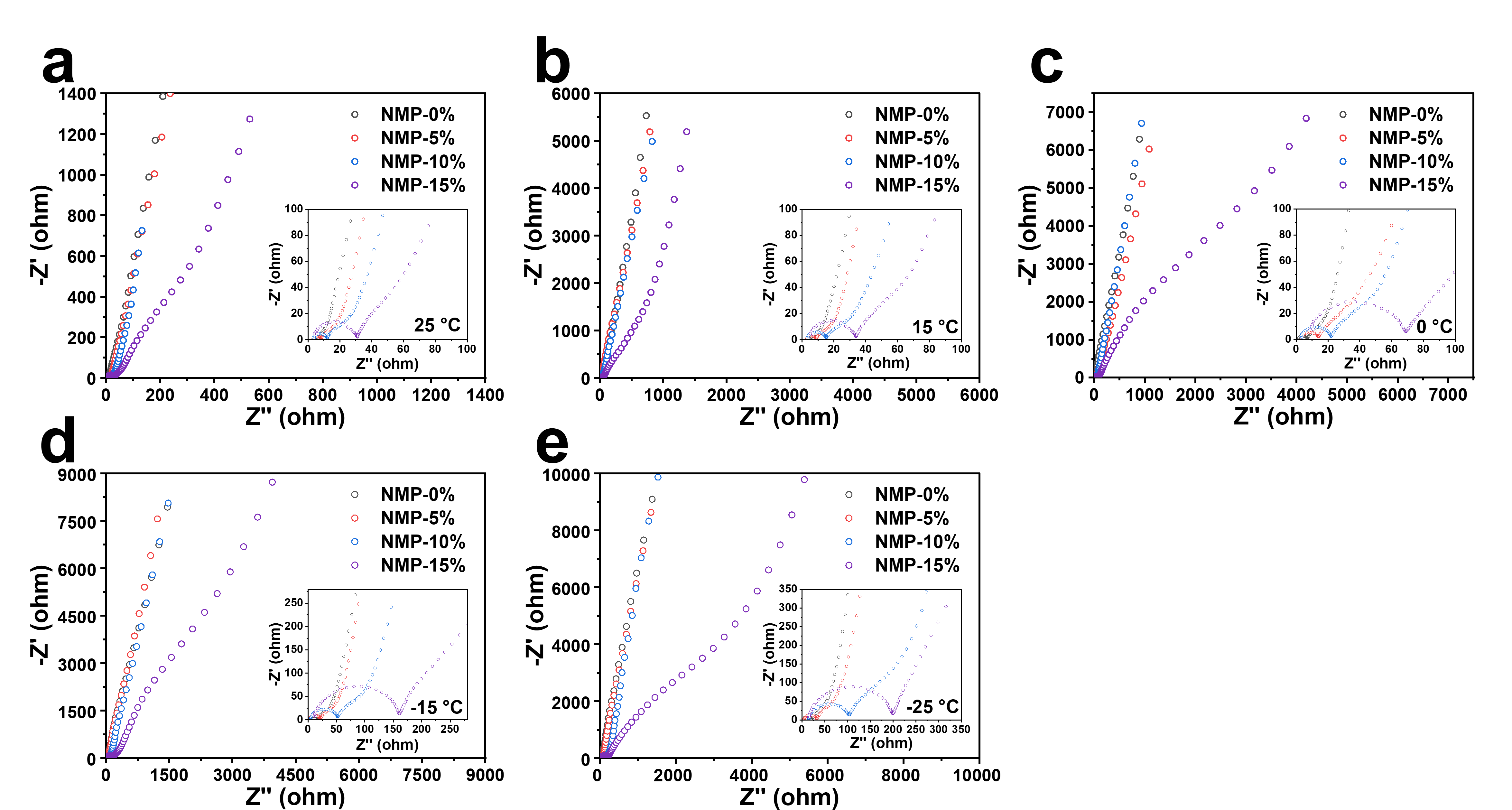


**Figure S17.** Impedance spectra of LEs with different NMP contents at different temperatures.

# In-situ infrared testing with disturbance voltage

The in-situ infrared spectroscopy test device consists of two symmetrical carbon cloth electrodes and a glass fiber separator. The configured LE-4.5 is fully soaked in the glass fiber separator, and then a constant voltage is applied through CHI 660e and FTIR is performed. As the disturbance voltage increases, the intensity of peaks in the 1500–1800 cm^−1^ region of the infrared spectrum slightly increases, with several peaks belonging to NMP becoming more pronounced (**Figure S18b**). On the contrary, the characteristic peaks mainly composed of water molecules in the 2800–4000 cm^−1^ region showed a slight shift (**Figure S18c**). This means that during the desolvation process, the content of NMP near the electrode increases and gradually enriches in IHP.


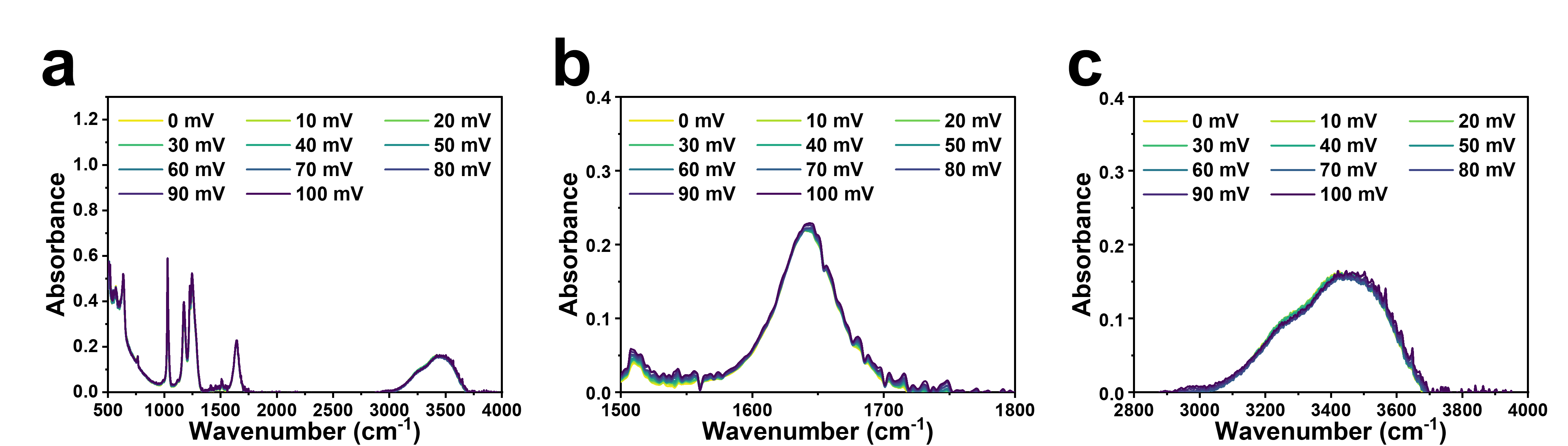


**Figure S18.** Changes in in-situ FTIR spectra under voltage disturbance. (a) Complete FTIR spectra under voltage disturbance. (b) FTIR spectrum absorption intensity under voltage disturbance in the range of 1500–1800 cm^−1^. (c) FTIR spectrum absorption intensity under voltage disturbance in the range of 2800–4000 cm^−1^.

# Analysis different types of NMP and water binding forms

As shown in **Figure S19,** the peaks of NMP-0% (pure H_2_O) and NMP-100% (pure NMP) are determined as the O−H bending vibration peak of H_2_O at 1635 cm^−1^ and the C=O stretching vibration peak of NMP at 1674 cm^−1^, respectively. NMP forms hydrogen bonds with water in a mixed solvent, the peak will appear region of 1635–1674 cm^−1^, which is mainly hydrogen-bonded NMP. In NMP-5%, the hydrogen-bonded NMP peak position is near 1637 cm^−1^. After adding LiOTf in solutions, the peak belongs to NMP or water shifts towards lower wavenumbers. The peak positions of pure H_2_O electrolyte and mixed electrolyte correspond to solvated water (1626 cm^−1^) and solvated NMP (1634 cm^−1^), respectively. Comparing the above peak position relationships, the following wavenumber relationship exists in LE: free NMP > hydrogen-bonded NMP > solvated NMP > solvated water.


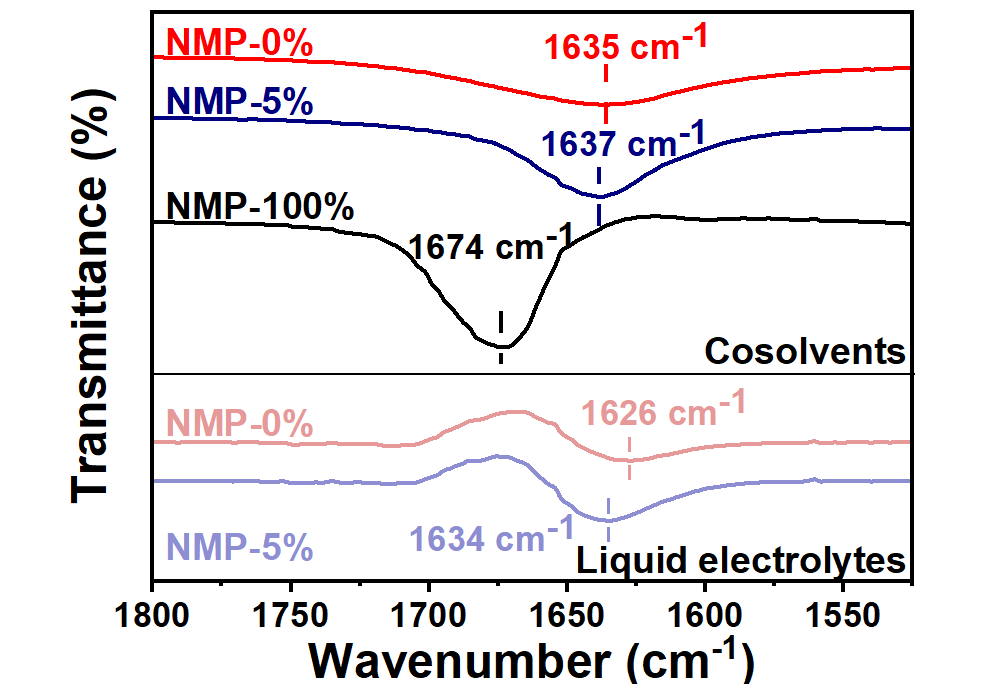


**Figure S19.** Main peak positions in different solutions at the range of 1530–1750 cm^−1^.


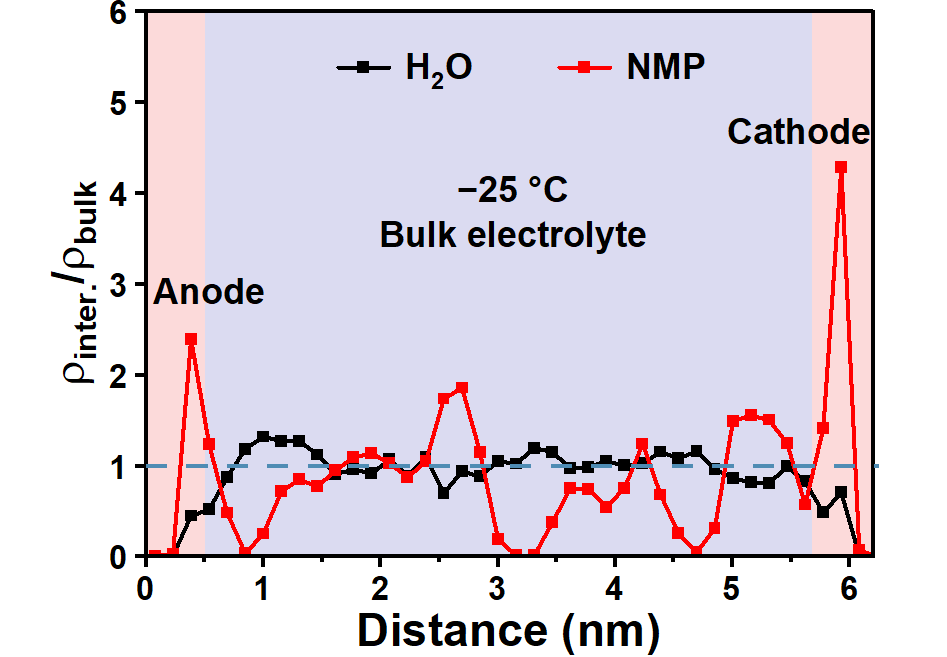


**Figure S20.** The MD result of NMP and H_2_O density distributions in OHE at −25 °C.


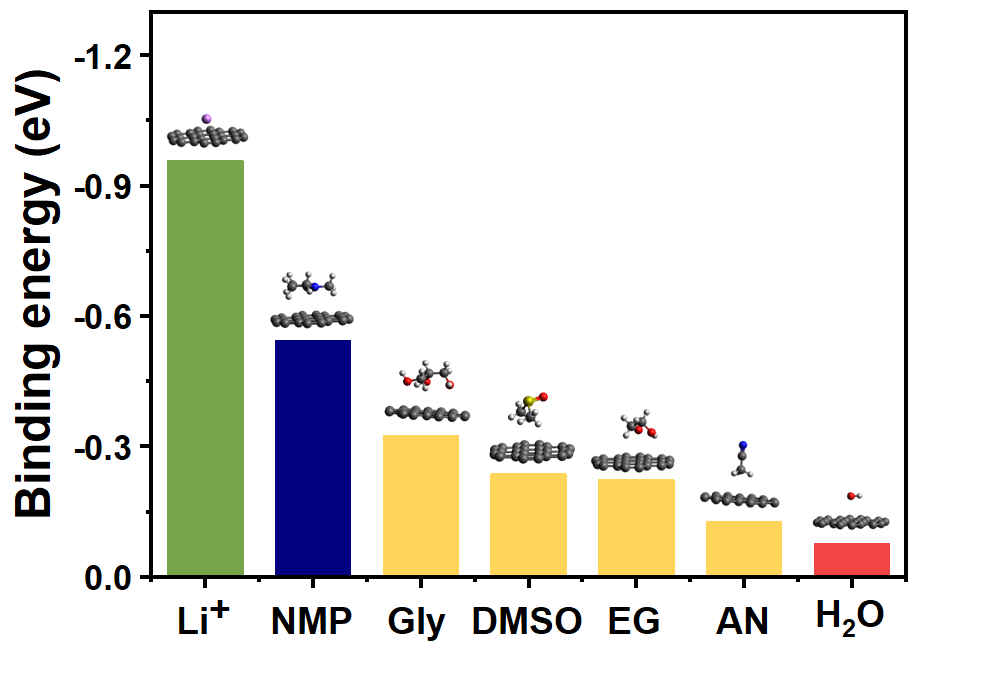


**Figure S21.** Specific adsorption of graphene and solvent molecules.


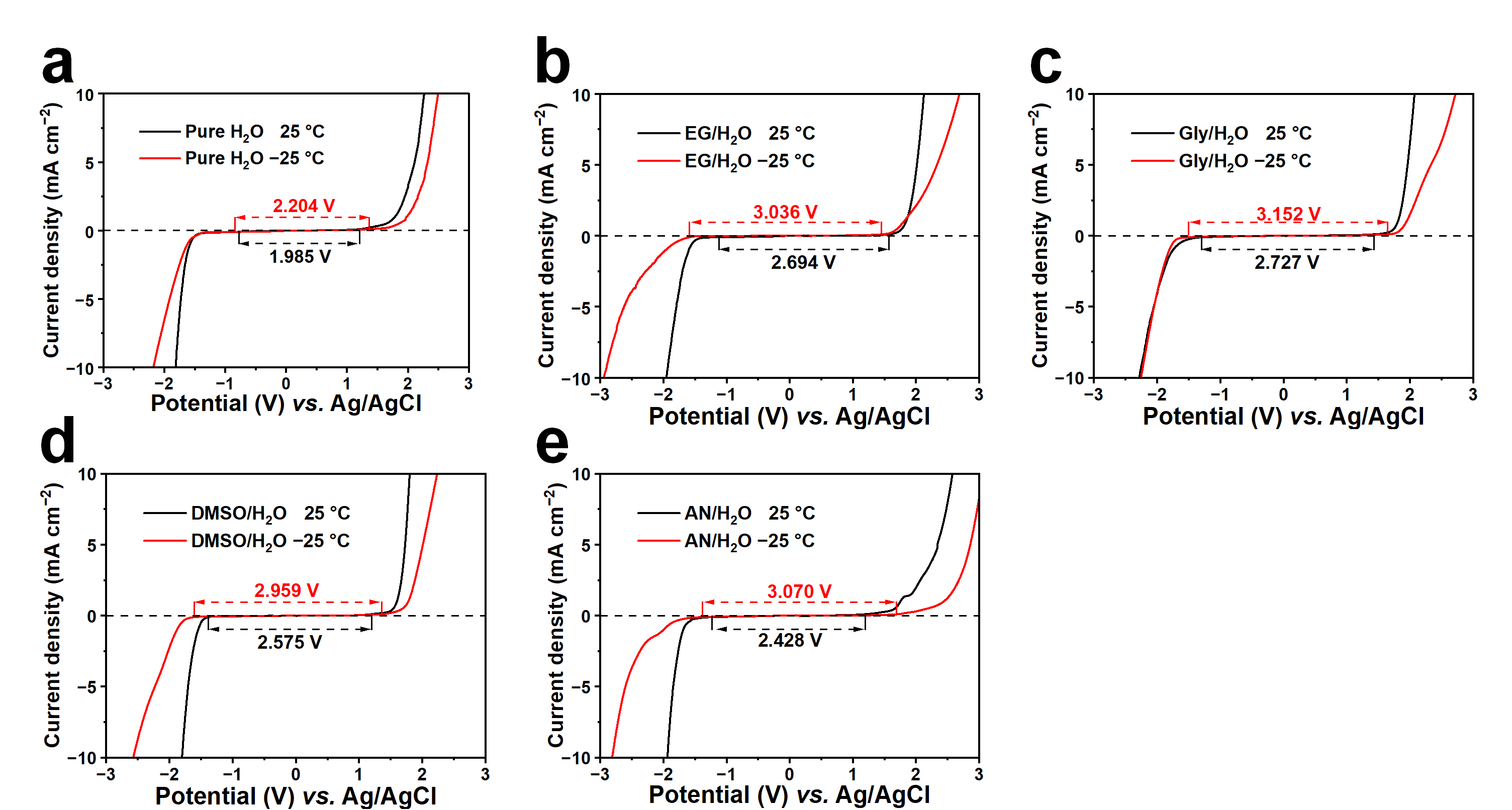


**Figure S22.** Changes with temperature of 4.5 M electrolyte ESW with and without organic solvent added. (a) Low temperature can broaden the ESW of pure H_2_O electrolyte. After adding organic solvents (b) EG, (c) Gly, (d) DMSO, and (e) AN, the ESW was further broadened at 25 °C and −25 °C.


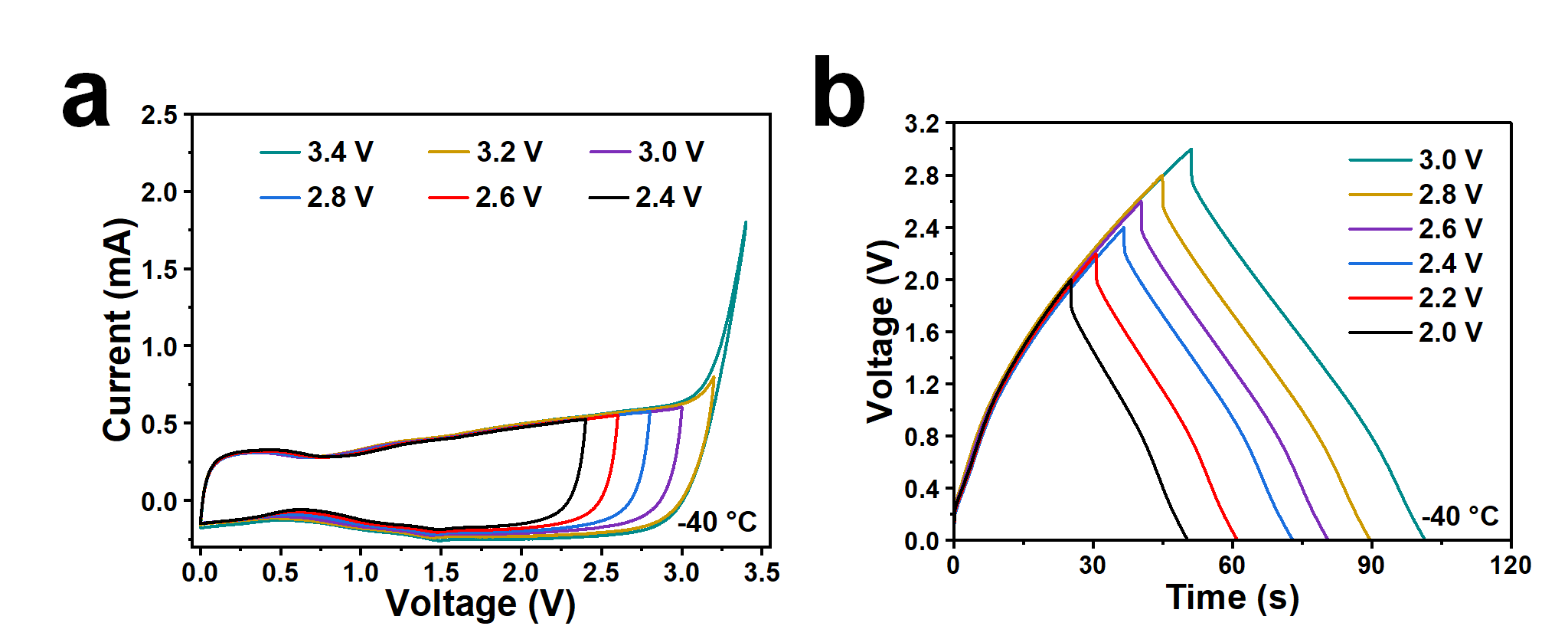


**Figure S23.** Explore the limiting output voltage of AFSC-4.5 at −40 °C. (a) CV curves of AFSC-4.5 under voltage from 0–2.4 V to 0–3.4 V. (b) GCD curves of AFSC-4.5 under output voltage from 2.0 V to 3.0 V.


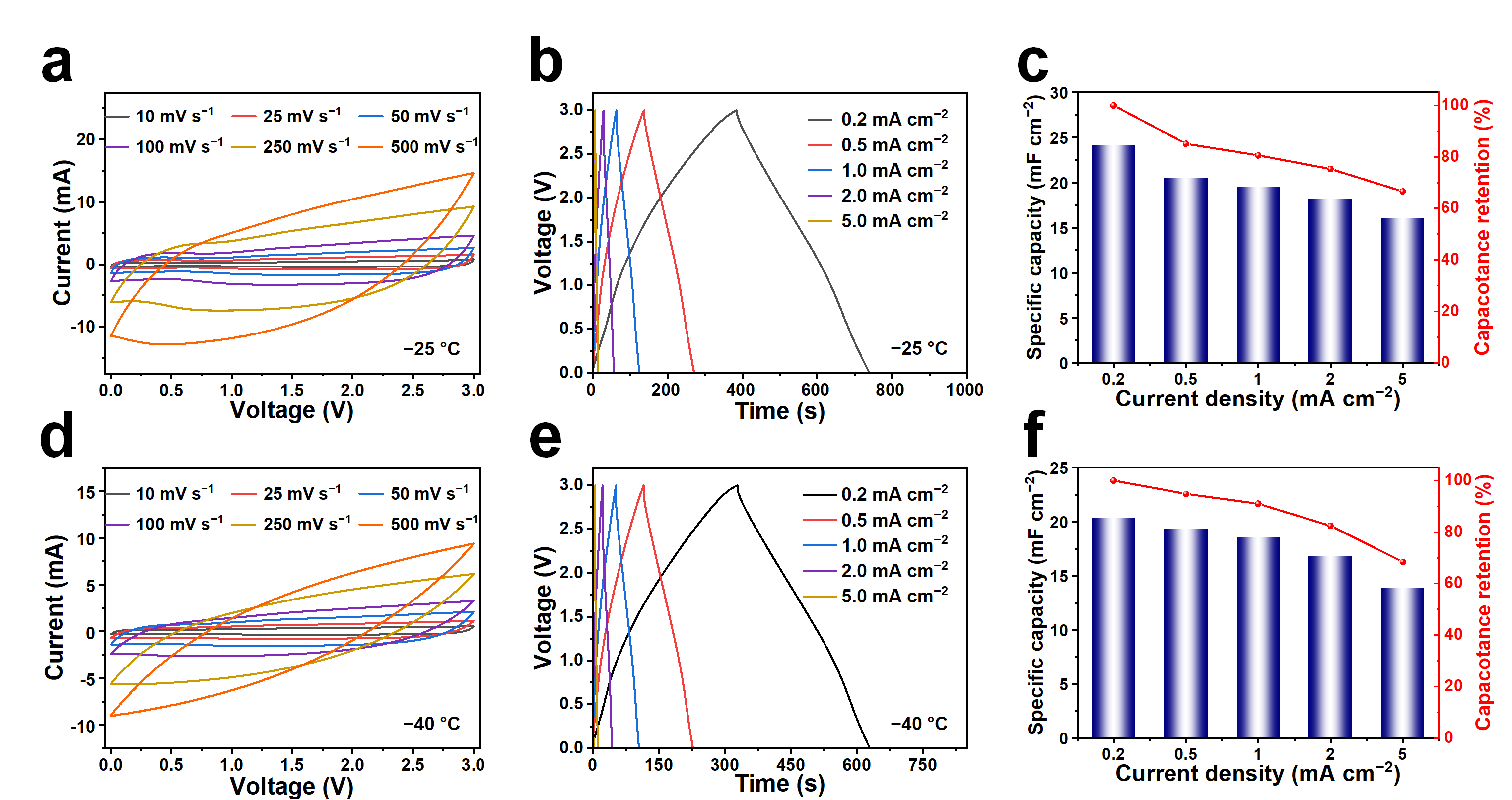


**Figure S24.** Electrochemical performance of AFSC-4.5 under 3.0 V output voltage at −25 and −40 °C. (a) The CV curves of AFSC-4.5 at −25 °C. (b) The GCD curves of AFSC-4.5 at −25 °C. (c) The rate performance of AFSC-4.5 at −25 °C. (d) The CV curves of AFSC-4.5 at −40 °C. (e) The GCD curves of AFSC-4.5 at −40 °C. (f) The rate performance of AFSC-4.5 at −40 °C. Excellent anti-freeze performance of OHE-4.5 allows AFSC-4.5 to maintain high capacity and rate performance under an astonishing 3.0 V output voltage.


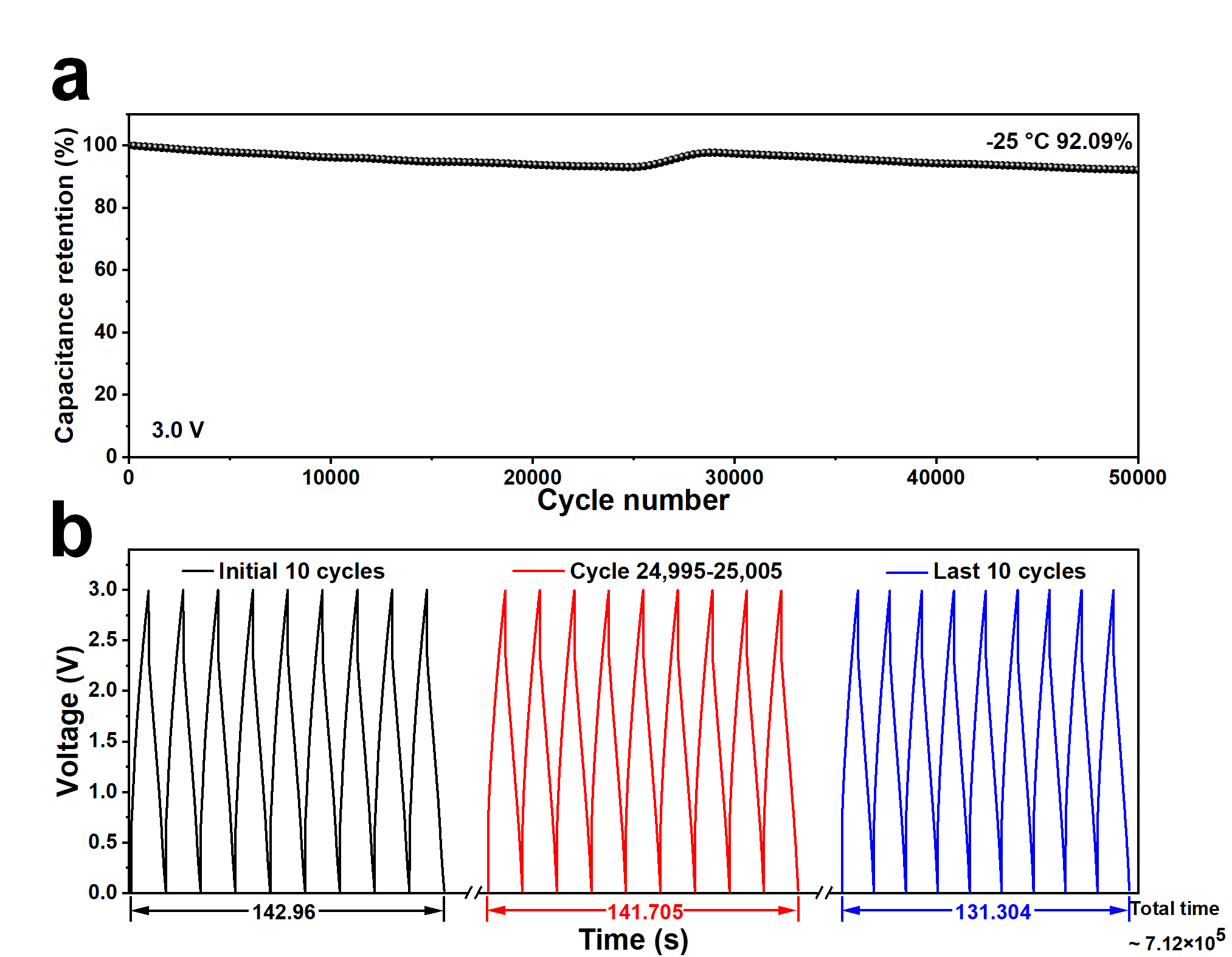


**Figure S25.** GCD stability test of AFSC-4.5 at −25 °C under 3.0 V voltage. (a) Stability of the AFSC-4.5 at −25 °C with 3.0 V voltage. (b) Detailed data from the GCD stability test (5.0 mA cm^−2^).


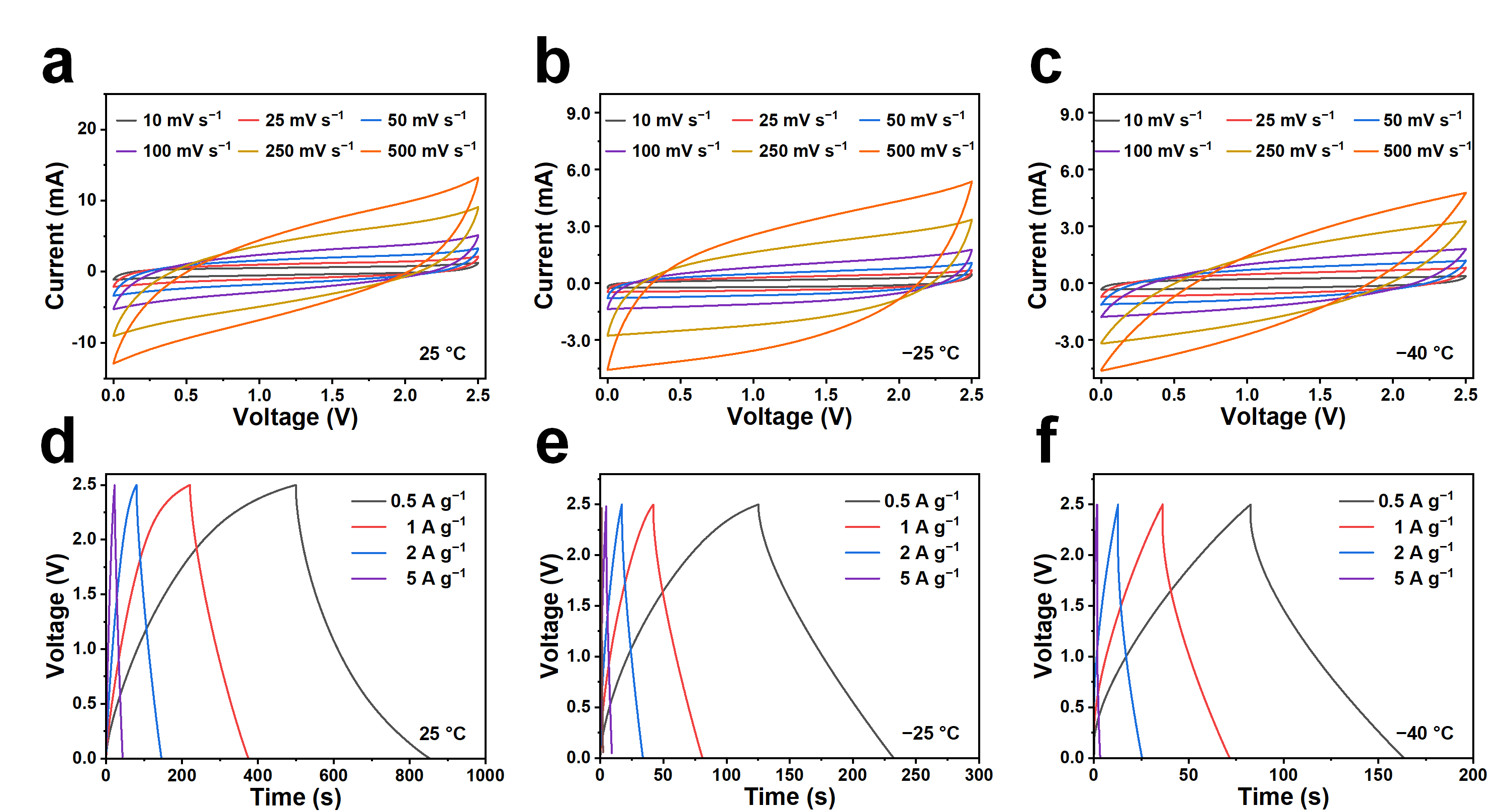


**Figure S26.** Electrochemical performance of AC-4.5 assembled by AC electrodes under 2.5 V output voltage at 25, −25, and −40 °C. (a, b, c) The CV curves of AC-4.5 under the output voltage of 2.5 V at 25, −25, and −40 °C. (d, e, f) GCD curves of AC-4.5 under the output voltage of 2.5 V at 25, −25, and −40 °C, respectively. Thanks to the interaction between OHE-4.5 molecules and the change of the IHP structure by NMP, AC-4.5 still has an output voltage of 2.5 V in a wide temperature range after replacing the electrode with AC.


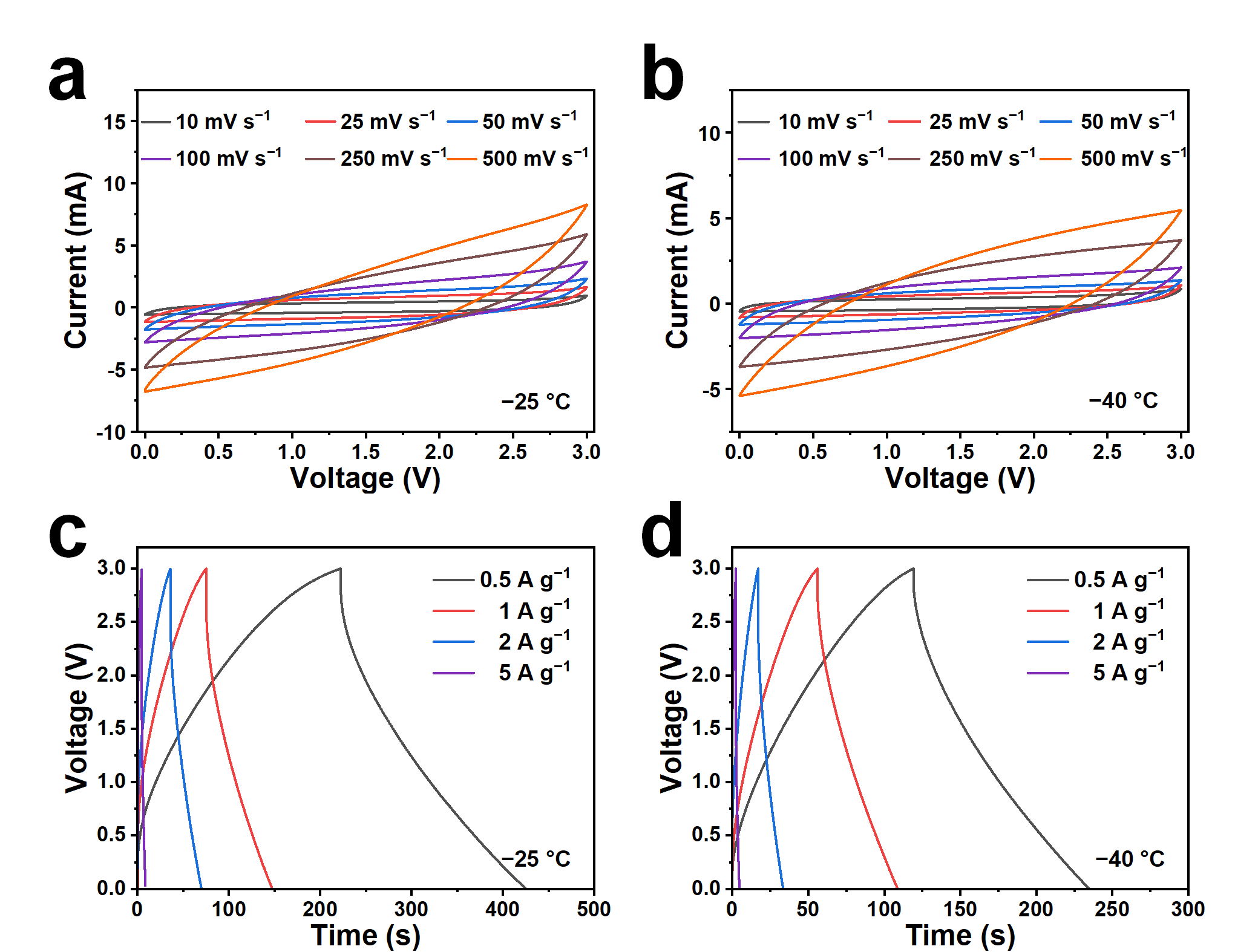


**Figure S27.** The CV curves and GCD curves of AC-4.5 under 3.0 V output voltage at −25 and −40 °C. (a, b) The CV curves of AC-4.5 under 3.0 V output voltage at −25 and −40 °C, respectively. (c, d) The GCD curves of AC-4.5 under 3.0 V output voltage at −25 and −40 °C, respectively. Similarly, the output voltage of AC-4.5 at low temperatures can be further expanded to 3.0 V.

**Table S1.** The comparison with the energy density of AFSC-4.5 at 25, −25, and −40 °C and the previously reported high-performance supercapacitors based on composite materials, conductive polymers, carbon materials, and material oxides add other organic co-solvent electrolytes at room temperature.

|  | Journal information | Temperature (°C) | Energy density (μWh cm^−2^) | Electrode materials | Specific capacity (mF cm^−2^) | Types |
| --- | --- | --- | --- | --- | --- | --- |
| 1 | This work | 25 | 22.05 | CNT | 25.40 | CNT |
|  |  | 0 | 17.54 |  | 21.18 |  |
|  |  | −15 | 16.87 |  | 19.43 |  |
|  |  | −25 | 15.61 |  | 17.92 |  |
|  |  | −40 | 13.55 |  | 15.61 |  |
| 2 | *Carbon Energy* **2022**, 4, 527-538. | 25 | 17.9 | PPY/carbon nanotube | 112.3 | Composite materials |
| 3 | ACS Appl. Mater. Interfaces **2020**, 12,  37977-37985. | 25 | 14.2 | MoN/CNTs | 285 | Composite materials |
| 4 | *Carbohydr. Polym.* **2021**, 274, 118667. | 25 | 9.3 | [SWCNT](https://www.sciencedirect.com/topics/chemistry/single-walled-nanotube) | 66.7 | Composite materials |
| 5 | Ceram. Int. **2022**, 48,  15721-15728. | 25 | 9.2 | PANI@Ti_3_C_2_T_x_ | 103.8 | Composite materials |
| 6 | *J. Power Sources* **2022**, 532, 231326. | 25 | 12.98 | PANI | 364 | Conductive polymers |
| 7 | *Chem. Eng. J.* **2023**, 455, 140949. | 25 | 12.51 | PANI | 140.75 | Conductive polymers |
| 8 | *J. Mater. Chem. A* **2018**, 6, 22858-22869. | 25 | 11.72 | PPy–3DrGO | 29.21 | Conductive polymers |
| 9 | *J. Energy Storage* **2022**, 50, 104231. | 25 | 9.4 | PPY | 212 | Conductive polymers |
| 10 | *J. Alloys Compd.* **2019**, 805, 1044-1051. | 25 | 6.94 | PPY | 58.8 | Conductive polymers |
| 11 | *Energy Environ. Sci.* **2021**, 14, 3075-3085. | 25 | 1.28 | PPY | 14.4 | Conductive polymers |
| 12 | *Adv. Energy Mater.* **2021**, 11, 2101523. | 25 | 7.0 | CNT | 9.5 | Carbon materials |
| 13 | *Polymer* 254, **2022**, 125109. | 25 | 5.8 | CNT | 9.5 | Carbon materials |
| 14 | ACS Appl. Energy Mater. **2022**, 5,  2211-2220. | 25 | 5.8 | PUCPM | 47 | Carbon materials |
| 15 | *J. Energy Chem.* **2022**, 72, 195-202. | 25 | 4.326 | AC | 12.1 | Carbon materials |
| 16 | *Adv. Energy Mater.* **2018**, 8, 1801840. | 25 | 3.49 | 3D graphene | 25.1 | Carbon materials |
| 17 | *Appl. Surf. Sci.* **2021**, 545, 149044. | 25 | 17.8 | WO_3_ | 50.06 | Material oxides |
| 18 | Energy Storage Mater. **2019**, 18, 397-404. | 25 | 9.0 | VN//MnO_2_ | 16.1 | Material oxides |
| 19 | Ceram. Int. **2020**, 46, 21736-21743. | 25 | 7.5 | MnO_2_/CFC | 54 | Material oxides |

**Table S2.** The energy density and power density of low temperature resistance supercapacitors at different temperatures.

|  | Journal Information | Temperature  (°C) | Energy  density  (μWh cm^−2^) | Power  density  (μW cm^−2^) |
| --- | --- | --- | --- | --- |
| 1 | This work | 25 | 22.05 | 1260.03 |
|  |  | −25 | 24.41 | 1560.99 |
|  |  | −40 | 23.16 | 1599.77 |
| 2 | *Chem. Eng. J.* **2023**, 455, 140949. | 25 | 3.96 | 734.55 |
|  |  | −40 | 3.50 | 492.97 |
| 3 | *Energy Environ. Sci.* **2021**, 14, 3075-3085. | 25 | 0.96 | 130.86 |
|  |  | −30 | 0.71 | 197.70 |
| 4 | *Adv. Energy Mater.* **2021**, 11, 2101523. | 25 | 7.10 | 1294.12 |
|  |  | –40 | 2.38 | 1861.57 |
| 5 | *J. Energy Chem.* **2022**, 72, 195-202. | 25 | 2.76 | 1134.03 |
|  |  | −30 | 5.09 | 1115.92 |
| 6 | *Nano Lett.* **2022**, 22, 6444-6453. | −35 | 13.21 | 1519.57 |
| 7 | Electrochem. Commun. **2023**, 148, 107456. | 25 | 6.97 | 1851.72 |
|  |  | −5 | 4.92 | 1978.12 |

**Table S3**. The comparison with the output voltage of AFSC-4.5 at 25, −25, and −40 °C and the previously reported output voltage of supercapacitors based on WIS or add other organic co-solvent electrolytes at room temperature.

|  | Journal Information | Electrolyte additives | Electrolyte  concentrations | Voltage  (V) |
| --- | --- | --- | --- | --- |
| 1 | AFSC-4.5  −40 °C–25 °C | Molar faction 4% NMP | 4.5 M LiOTf | 2.5 |
|  | AFSC-4.5  −40 °C–−25 °C | Molar faction 4% NMP | 4.5 M LiOTf | 3.0 |
| 2 | Electrochim. Acta **2021**,  376, 137984. | 22 wt% DMSO in electrolyte | m_Na2SO4_ : m_toal_  = 22% | 1.8 |
| 3 | J. Mater. Chem. A **2021**, 9,  12051-12059. | v_DMSO_ : v_water_  1:24 | LiCl  3 M | 1.0 |
| 4 | J. Power Sources **2022**,  532, 231326. | 16 wt% DMSO in PVA/D-F1 | 0.5 M (NH_4_)_2_SO_4_ | 0.8 |
| 5 | *Nano Lett.* **2020**, 20,  1907-1914. | n_DMSO_ : n_water_  1 : 1 | 2 M H_2_SO_4_ | 0.8 |
| 6 | *Adv. Energy Mater.* **2021,** 11, 2101523. | V_EG_ : V_water_  1 : 1 | 10.5 M LiTFSI | 2.3 |
| 7 | J. Power Sources **2022**, 528,  231210. | V_EG_ : V_water_  1 : 9 | 1 M ZnCl_2_  3 M NH_4_Cl | 1.8 |
| 8 | *J. Energy Chem.* **2022,** 72, 195-202. | V_EG_ : V_water_  1 : 1 | 10 M LiCl | 1.6 |
| 9 | Carbohydr. Polym. **2021,** 274,  118667. | V_EG_ : V_water_  6 : 4 | 2.5 M NaCl | 1.6 |
| 10 | Adv. Energy Mater. **2018,** 8,  1801967. | m_EG_ : m_water_  2 : 1 | 1 m LiCl | 1.0 |
| 11 | *Polymer* 254, **2022,** 125109. | m_Gly_ : m_water_  3 : 4 | 1.8 g CaCl_2_ in 7 g solution | 2.1 |
| 12 | ACS Appl. Energy Mater. **2020**, 3, 1944-1951. | V_Gly_ : V_water_  1 : 2 | 2 M LiClO_4_ | 1.8 |
| 13 | ACS Appl. Mater. Interfaces **2020**, 12, 56393-56402. | m_Gly_ : m_water_  1 : 10 | 2 M NaCl | 1.0 |
| 14 | *Small* 17, **2021**, 2103091. | V_Gly_ : V_water_  1 : 2 | 0.1 mol LiCl in 30 mL solution | 1.0 |
| 15 | *J. Mater. Chem. A* **2021**, *9*, 18406-18420. | m_Gly_ : m_water_  13 : 15 | 1.2 g CaCl_2_ | 0.8 |
| 16 | ACS Appl. Energy Mater. **2020**, 3, 3692-3703. | Pure Water | 6 g LiCl in  60 mL water, | 1.3 |
| 17 | *Materials Horizons* **2017**, 4, 1145-1150. | Pure Water | 1.0 M H_2_SO_4_ | 1.0 |
| 18 | Carbon Energy **2022**, 4,  527-538. | Pure Water | 0.3 g LiCl | 0.8 |
| 19 | ACS Appl. Mater. Interfaces **2020**, 12,  37977-37985. | Pure Water | 1 M H_2_SO_4_ | 0.6 |
| 20 | *Chem. Eng. J.* **2023**, 455, 140949. | Pure Water | 1 M H_2_SO_4_ | 0.6 |
| 21 | *Chem. Mater.* **2016**, 28, 3944-3950. | WIS | 5 M LiTFSI | 2.4 |
| 22 | *J. Mater. Chem. A* **2019**, 7, 15801-15811. | WIS | 7 m LiTFSI | 2.3 |
| 23 | *J. Mater. Chem. A* **2019**, 7, 24800-24806 | WIS | 10 M LiTFSI | 2.2 |

**Table S4.** Specific capacity, energy density and power density of AFSC-4.5 at different temperatures.

|  | Voltage  (V) | Temperature  (°C) | Specific capacity  (mF cm^−2^) | Energy density  (μWh cm^−2^) | Power density  (μW cm^−2^) |
| --- | --- | --- | --- | --- | --- |
| AFSC-4.5 | 2.5 | 25 | 25.40 | 22.05 | 1260.03 |
|  |  | 0 | 21.17 | 18.38 | 1267.65 |
|  |  | −15 | 19.43 | 16.87 | 1283.95 |
|  |  | −25 | 17.93 | 15.56 | 1296.73 |
|  |  | −40 | 15.61 | 13.55 | 1374.05 |
|  | 3.0 | −25 | 19.53 | 24.41 | 1560.98 |
|  |  | −40 | 18.53 | 23.16 | 1599.77 |
